# Long-Term Effects of a Novel Continuous Remote Care Intervention Including Nutritional Ketosis for the Management of Type 2 Diabetes: A 2-Year Non-randomized Clinical Trial

**DOI:** 10.3389/fendo.2019.00348

**Published:** 2019-06-05

**Authors:** Shaminie J. Athinarayanan, Rebecca N. Adams, Sarah J. Hallberg, Amy L. McKenzie, Nasir H. Bhanpuri, Wayne W. Campbell, Jeff S. Volek, Stephen D. Phinney, James P. McCarter

**Affiliations:** ^1^Virta Health Corp, San Francisco, CA, United States; ^2^Indiana University Health Arnett, Lafayette, IN, United States; ^3^Department of Nutrition Science, Purdue University, West Lafayette, IN, United States; ^4^Department of Human Sciences, The Ohio State University, Columbus, OH, United States; ^5^Department of Genetics, Washington University School of Medicine, St. Louis, MO, United States

**Keywords:** type 2 diabetes, nutritional ketosis, HbA1c, body composition, reversal and remission

## Abstract

**Purpose:** Studies on long-term sustainability of low-carbohydrate approaches to treat diabetes are limited. We previously reported the effectiveness of a novel digitally-monitored continuous care intervention (CCI) including nutritional ketosis in improving weight, glycemic outcomes, lipid, and liver marker changes at 1 year. Here, we assess the effects of the CCI at 2 years.

**Materials and methods:** An open label, non-randomized, controlled study with 262 and 87 participants with T2D were enrolled in the CCI and usual care (UC) groups, respectively. Primary outcomes were retention, glycemic control, and weight changes at 2 years. Secondary outcomes included changes in body composition, liver, cardiovascular, kidney, thyroid and inflammatory markers, diabetes medication use and disease status.

**Results:** Reductions from baseline to 2 years in the CCI group resulting from intent-to-treat analyses included: HbA1c, fasting glucose, fasting insulin, weight, systolic blood pressure, diastolic blood pressure, triglycerides, and liver alanine transaminase, and HDL-C increased. Spine bone mineral density in the CCI group was unchanged. Use of any glycemic control medication (excluding metformin) among CCI participants declined (from 55.7 to 26.8%) including insulin (-62%) and sulfonylureas (-100%). The UC group had no changes in these parameters (except uric acid and anion gap) or diabetes medication use. There was also resolution of diabetes (reversal, 53.5%; remission, 17.6%) in the CCI group but not in UC. All the reported improvements had *p* < 0.00012.

**Conclusion:** The CCI group sustained long-term beneficial effects on multiple clinical markers of diabetes and cardiometabolic health at 2 years while utilizing less medication. The intervention was also effective in the resolution of diabetes and visceral obesity with no adverse effect on bone health.

**Clinical Trial Registration:** Clinicaltrials.gov NCT02519309

## Introduction

Type 2 diabetes (T2D), obesity, and metabolic disease impact over one billion people and present a challenge to public health and economic growth (1, 2). In the United States, over 30 million people have diabetes and it is recognized among the leading causes of morbidity and mortality, especially through increased cardiovascular disease (CVD) (3). The remission rate under usual care is 0.5–2% (4) while an intensive lifestyle intervention resulted in remission rates (both partial and complete) of 11.5 and 9.2% at 1 and 2 years (5). When lifestyle interventions are insufficient, medications are indicated to manage the disease and slow progression (6, 7).

When T2D care directed at disease reversal is successful, this includes achievement of restored metabolic health, glycemic control with reduced dependence on medication, and in some cases disease remission. Three non-pharmaceutical approaches have demonstrated high rates of at least temporary T2D diabetes reversal or remission: bariatric surgery, very low calorie diets (VLCD), and nutritional ketosis achieved through carbohydrate restriction (8–10). In controlled clinical trials, each approach has demonstrated improved glycemic control and CVD risk factors, reduced pharmaceutical dependence, and weight loss. The three approaches show a similar time-course with glycemic control preceding weight loss by weeks or months, suggesting potential overlap of mechanisms (11, 12).

With bariatric surgery, up to 60% of patients demonstrate T2D remission at 1 year (13). Outcomes at 2 years and beyond indicate ~50% of patients can achieve ongoing diabetes remission (13, 14). The second Diabetes Surgery Summit recommended using bariatric surgery to treat T2D with support from worldwide medical and scientific societies (15), but both complications associated with surgery and cost limit its widespread use (16, 17). VLCDs providing <900 kcal/day allow rapid discontinuation of most medications, improved glycemic control, and weight loss. This approach is necessarily temporary, however, with weight regain and impaired glucose control typically occurring within 3–6 months of reintroduction of substantial proportions of dietary carbohydrates (9, 18–20).

A third approach to diabetes reversal is sustained dietary carbohydrate restriction. Low-carbohydrate diets have consistently elicited improvements in T2D, metabolic disease, and obesity up to one year (21, 22), however, longer-term studies and studies including patients prescribed insulin are limited. A low-carbohydrate Mediterranean diet caused remission in 14.7% of newly diagnosed diabetes patients at 1 year vs. 4.1% with a low-fat diet (23), and a small randomized trial utilizing a ketogenic diet demonstrated improved weight and diabetes control at 1 year (24). Systematic reviews also corroborate the effectiveness of a low-carbohydrate diet for T2D (25, 26) and it has recently become a consensus recommended dietary option (27–29). Nonetheless, sustained adherence to carbohydrate restriction is considered challenging (27, 28) and an LDL-C increase is sometimes observed (30–33). Given that total LDL-particles (LDL-P), small LDL-P, and ApoB tend to improve or remain unchanged, the impact of an increase in LDL-C on CVD risk in the context of this dietary pattern is unknown.

We have previously reported 1 year outcomes of an open-label, non-randomized, controlled, longitudinal study with 262 continuous care intervention (CCI), and 87 usual care (UC) participants with T2D (10). The CCI included individualized support with telemedicine, health coaching, and guidance in nutritional ketosis using an individualized low-carbohydrate diet. Nutritional guidance encouraged sustained nutritional ketosis; patients were counseled on preparation of a low-carbohydrate diet adapted to meet their life circumstances. Eighty-three percent of CCI participants remained enrolled at 1 year and 60% of completers achieved an HbA1c <6.5% while prescribed metformin or no diabetes medication. Weight was reduced and most CVD risk factors improved (33).

Long-term studies of low-carbohydrate dietary approaches to treat type 2 diabetes and obesity are limited, particularly among those that are delivered and supported remotely. Here we assess longer-term outcomes in CCI participants with T2D at 2 years, as well as the effects on body composition and related comorbidities. The primary aims were to investigate the effect of the CCI on retention, glycemic control, diabetes status, and weight. Secondary aims included: (1) investigating the effect of the CCI on bone mineral density, visceral fat composition, cardiovascular risk factors, liver, kidney, thyroid and inflammatory markers, and related disease outcomes (e.g., metabolic syndrome); and (2) comparing 2-year outcomes between the CCI and UC groups.

## Materials and Methods

### Study Design and Participants

The comprehensive study design was previously published with the 1 year outcomes (10, 33), and the results presented here are the follow-up 2-year results from the same ongoing five-year clinical trial (*Clinical trials.gov identifier: NCT02519309*). This is an open-label, non-randomized, outpatient study, and results presented here are based on data from the first 2 years of the trial collected from August, 2015 to May, 2018. Participants aged 21 to 65 years with a confirmed diagnosis of T2D and a body mass index (BMI) >25 kg/m^2^ self-selected to receive either the CCI or usual care (UC). Major exclusion criteria are listed in the previous publication (10, 33). All study participants provided written informed consent and the study was approved by the Franciscan Health Lafayette Institutional Review Board, Lafayette, IN, USA.

### Study Interventions

#### Continuous Care Intervention (CCI)

For the intervention group, participants were advised to achieve and sustain nutritional ketosis (blood BHB level of 0.5–3.0 mmol L^−1^) through sufficient carbohydrate restriction (initially <30 g day^−1^ but gradually increased based on personal carbohydrate tolerance and health goals). Participants' daily protein intake was initially targeted at a level of 1.5 g kg^−1^ of a medium-frame ideal weight body and further individualized based on biomarkers. Participants were instructed to include sufficient dietary fat in meals to achieve satiety without tracking energy intake. Nutrition education directed consumption of monounsaturated and saturated fat with sufficient intake of omega-3 and omega-6 polyunsaturated fats. The participants were also encouraged to consume sufficient fluid, vitamins and minerals including sodium and magnesium, especially if signs of mineral deficiency were encountered (e.g., decreased circulating volume) (10).

The CCI participants were provided access to a web-based software application (app), which was used to provide telemedicine communication, online resources and biomarker tracking tools. The participants used the app to upload and monitor their reportable biomarkers including body weight, blood glucose and beta-hydroxybutyrate (BHB). Biomarkers allowed for daily feedback to the care team and individualization of patient instruction. Frequency of reporting was personalized over time based on care needs. The web-based app was also used by participants to communicate with their remote care team consisting of a health coach and a medical provider. The remote care team provided education and support regarding dietary changes, behavior modification techniques for maintenance of lifestyle changes, and directed medication changes for diabetes and antihypertensive medications. Education modules covered core concepts related to the dietary changes for achieving nutritional ketosis, and adaptation to and maintenance of the diet (10). Participants selected their preferred education mode (CCI-virtual, *n* = 126 or CCI-onsite, *n* = 136) during recruitment. The CCI-virtual group received care and education primarily via app-based communication. The CCI-onsite group also received care and education via clinic-based group meetings (weekly for 12 weeks, bi-weekly for 12 weeks, monthly for 6 months, and then quarterly in the second year). All participants had access to the app for communication with their care team, online resources, biomarker tracking and the opportunity to participate in an online peer community for social support.

#### Usual Care (UC)

The participants recruited for usual care (UC) received care from their primary care physician or endocrinologist and were counseled by a registered dietician as part of a diabetes education program. These participants received the American Diabetes Association (ADA) recommendations on nutrition, lifestyle and diabetes management. No modification of their care was made for the study and routine biomarkers (weight, glucose and ketones) were not collected from these participants. This group was used as a reference control to study the effect of disease progression over 2 years in a cohort of participants prospectively recruited from the same geography and healthcare system.

**Figure 1:**
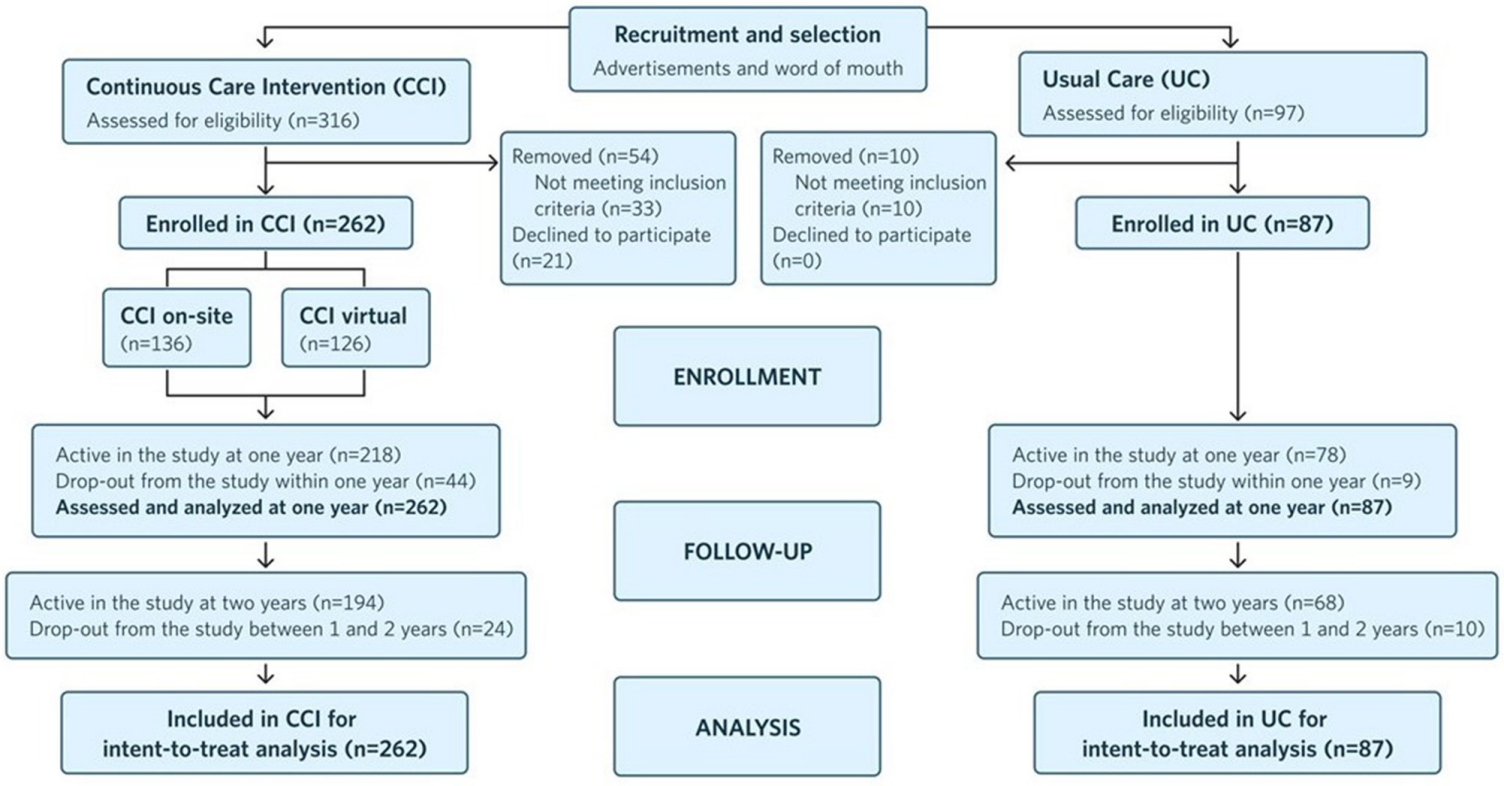
Flow chart of participants in each stage of the study from recruitment to 2 years post-enrollment and analysis.

Figure 1 depicts the study flow from recruitment to 2 years post-enrollment.

### Outcomes

#### Primary Outcomes

The primary outcomes were retention, HbA1c, HOMA-IR derived from insulin or c-peptide (formulas listed in Supplementary Table 1), fasting glucose, fasting insulin, c-peptide and weight.

#### Secondary Outcomes

Long-term body composition changes assessed in CCI participants included bone mineral density (BMD), abdominal fat content (CAF and A/G ratioC), and lower extremity lean mass (LELM). Body composition was not assessed in UC participants. Cardiovascular-related markers included resting blood pressure (systolic and diastolic), triglycerides, total cholesterol, HDL-C and calculated LDL-C (Friedewald equation, Supplementary Table 1). Liver-related markers included the liver enzymes alanine aminotransferase (ALT), aspartate aminotransferase (AST), and alkaline phosphatase (ALP), bilirubin, and two calculated liver scores: non-alcoholic liver fat score (NLF) and non-alcoholic liver fibrosis score (NFS) (formulas in Supplementary Table 1). Kidney-related markers included serum creatinine, uric acid, anion gap, blood urea nitrogen (BUN), and estimated glomerular filtration rate (eGFR). Thyroid-related markers included thyroid stimulating hormone (TSH) and free T4. Inflammatory markers included high sensitive C-reactive protein (hsCRP) and white blood cell count (WBC). Changes in overall diabetes medication use, use by class, and insulin dose were tracked over the 2 years of the trial.

The prevalence and resolution of T2D (diabetes reversal, partial and complete remission), metabolic syndrome, liver steatosis, and fibrosis were evaluated at baseline and 2 years using the criteria provided in Supplementary Table 2. Assignment of metabolic syndrome was based on the presence of three of the five defined criteria according to measured laboratory and anthropometric variables (34, 35) and pharmacological treatment for any of the conditions was not considered in the assignment (Supplementary Table 2).

Adverse events encountered in the study were reported to the Principal Investigator and reviewed by the Institutional Review Board (IRB).

### Laboratory Measures

Clinical anthropometrics and laboratory blood analyte measurements were obtained at baseline, 1 year, and 2 years from the CCI and UC participants. Details of the methods were previously published (10). All blood analytes were measured at a Clinical Laboratory Improvement Amendments (CLIA) certified laboratory.

### Body Composition Measures

The CCI participants' total body composition was measured at baseline, 1 year and 2 years using dual-energy X-ray absorptiometry (DXA) (Lunar GE Prodigy, Madison, WI). Participants were scanned while wearing light clothing using standard clinical imaging procedures. The scans obtained were analyzed using GE Encore software (v11.10, Madison, WI). In many obese patients, full body scans were not obtained due to the scanner not accommodating the patient's complete body resulting in issues such as cropping of the arms and/or overlapping of arms with the chest (36, 37). To address these limitations, changes in bone density and fat and lean mass were assessed using subregions rather than the full body scan. We assessed changes in the bone mass by evaluating total spine bone mineral density (BMD) from baseline to 2 years (38). For assessment of fat mass, we manually selected the central abdominal fat (CAF) region using the software and evaluated the changes in CAF over time, as previously suggested for overweight individuals (36, 39). Furthermore, we assessed changes in the android:gynoid (A/G) ratio by time. Due to lack of proper arm lean mass measurement, we analyzed the lower extremity lean mass (LELM) to assess weight-related changes in lean mass over time (40, 41).

### Statistical Analyses

All analyses were conducted using SPSS statistical software (Version 25.0, Armonk, NY). First, we examined the assumptions of normality and linearity. According to Kline's (2011) guidelines (42), 14 outcomes (i.e., fasting insulin, insulin and C-peptide-derived HOMA-IR scores, triglycerides, ALT, AST, bilirubin, N-LFS, BUN, serum creatinine, TSH, Free T4, hsCRP, and BHB) were positively skewed. We explored two approaches to handling the skewed variables: natural log-transformations and removing the top 1% of values. For N-LFS, which includes both positive and negative values, a modulus log-transformation was performed instead of a natural log-transformation (43). For most variables, both approaches resulted in new skew and kurtosis values within the acceptable range. One variable (triglycerides) was only corrected via log-transformation, whereas two variables (C-peptide-derived HOMA-IR and TSH) were only corrected by removing the top 1% of values. For the other variables, we conducted sensitivity analyses to compare the two approaches. Because the results did not differ between the approaches and because interpretation of outcomes is more difficult with transformed variables, we report results from the approach of removing the top 1% of values for all variables except triglycerides. For triglycerides, analyses were performed and *p*-values reported on the log-transformed variable but the means and standard errors reported were computed from the untransformed variable. Next, we ran independent sample *t*-tests to examine differences in baseline characteristics between CCI and UC, and completers and dropouts.

We performed linear mixed-effects models (LMMs) to assess (1) within-group changes in the continuous study outcomes from baseline to 2 years and (2) between-group differences (CCI vs. UC) in the study outcomes at 2 years. The LMMs included fixed effects for time, group (CCI vs. UC), and a time by group interaction. Covariates included baseline age, sex, race (African American vs. other), BMI, and insulin use. This maximum likelihood-based approach uses all available repeated data, resulting in an intent-to-treat analysis. An unstructured covariance structure was specified for all models to account for correlations between repeated measures.

Within-group changes and between-group differences in dichotomous disease outcome variables [i.e., diabetes reversal, diabetes remission (partial or complete) and complete remission (44), metabolic syndrome (34, 35), steatosis (45), fibrosis (46)] were assessed, controlling for baseline age, sex, race, time since diagnosis, BMI, and insulin use. For this set of analyses, multiple imputation was used to replace missing values from baseline and 2 years with a set of plausible values, facilitating an intent-to-treat analysis (all ns = 262). Missing values were estimated from 40 imputations (47) from logistic regression. Within-group changes from baseline to 2 years and between-group differences at 2 years were assessed using generalized estimating equations with binary logistic models and unstructured covariance matrices.

We also examined changes in participants' diabetes medication use. First, we compared the rates of diabetes medication use within groups from baseline to 2 years using McNemar's test with continuity correction when appropriate. Next, we calculated the proportion of participants in each group with each diabetes medication class eliminated, reduced, not changed, increased, or added. Paired *t*-tests were used to assess within-group changes in insulin dosages from baseline to 2 years among participants taking insulin at baseline and among participants taking insulin at both baseline and 2 years.

We conducted a second set of analyses with 2-year completers only. Results of the completers-only analyses appear in Supplementary Table 3. Given that 2 different modes (virtual and onsite) were utilized for delivery of the CCI group educational content, we also conducted another set of analyses to assess whether differences existed between the groups on all analyses of primary outcomes. As in our prior time points (10, 48), no group differences were found; thus, the data from the two CCI educational groups were combined for this report. For all study analyses, nominal significance levels (P) are presented in the tables. A significance level of *P* < 0.0012 ensures overall simultaneous significance of *P* < 0.05 over the 43 variables using Bonferroni correction.

## Results

### Participant Characteristics

Table 1 presents baseline characteristics of the 262 CCI and 87 UC participants. Participants did not differ between groups in demographic characteristics, except the proportion of African Americans was higher in the CCI group. Baseline characteristics were well-matched between the groups, except for mean weight and BMI, which were higher in the CCI group. There were no differences between completers and dropouts on baseline characteristics for either group.

**Table 1:** Baseline characteristics.

	**All**		**Completers with data**		**Dropout or missing data**		**Completers- Dropouts**
	***N***	**Mean (SD) or ± SE**	***N***	**Mean (SD) or ± SE**	***N***	**Mean (SD) or ± SE**	**Mean ± SE**
**Age (years)**							
CCI-all education	262	53.8 (8.4)	194	54.4 (8.2)	68	51.9 (8.7)	2.5 ± 1.2
Usual Care	87	52.3 (9.5)	68	51.4 (9.4)	19	55.6 (9.5)	−4.2 ± 2.4
CCI-all vs. usual care		1.4 ± 1.1		3.0 ± 1.2		−3.6 ± 2.4	
**African American (%)**							
CCI-all education	262	6.9 ± 1.6	194	6.2 ± 1.7	68	8.8 ± 3.5	−2.6 ± 3.6
Usual Care	87	0.0 ± 0.0	68	0.0 ± 0.0	19	0.0 ± 0.0	—
CCI-all vs. usual care		6.9 ± 1.6^*^		6.2 ± 1.7^*^		8.8 ± 3.5	
**Body mass index (kg m**^**−2**^**)**							
CCI-all education	257	40.42 (8.81)	190	40.41 (8.42)	67	40.46 (9.90)	−0.05 ± 1.25
Usual Care	83	36.72 (7.26)	64	36.90 (7.41)	19	36.11 (6.89)	0.79 ± 1.91
CCI-all vs. usual care		3.70 ± 1.07^*^		3.51 ± 1.18		4.34 ± 2.43	
**Female (%)**							
CCI-all education	262	66.79 ± 2.92	194	65.98 ± 3.41	68	69.12 ± 5.64	−3.14 ± 6.66
Usual Care	87	58.62 ± 5.31	68	60.29 ± 5.98	19	52.63 ± 11.77	7.66 ± 12.90
CCI-all vs. usual care		8.17 ± 6.06		5.69 ± 6.76		16.49 ± 12.35	
**Waist circumference (in)**							
CCI-all education	218	49.02 (5.64)	159	49.04 (6.40)	59	48.97 (6.89)	0.06 ± 1.00
Usual Care	83	46.41 (5.64)	64	46.33 (5.63)	19	46.67 (5.82)	0.34 ± 1.48
CCI-all vs. usual care		2.61 ± 0.81		2.71 ± 0.92		2.30 ± 1.75	
**Years since type 2 diabetes diagnosis**							
CCI-all education	261	8.44 (7.22)	193	8.15 (7.02)	68	9.25 (7.75)	−1.1 ± 1.02
Usual Care	71	7.85 (7.32)	63	7.90 (7.41)	8	7.38 (7.05)	0.53 ± 2.77
CCI-all vs. usual care		0.59 ± 0.97		0.25 ± 1.03		1.88 ± 2.87	
**GLYCEMIC**							
**Hemoglobin A1c (%)**							
CCI-all education	262	7.6 (1.5)	194	7.5 (1.41)	68	7.9 (1.7)	−0.4 ± 0.2
Usual Care	87	7.6 (1.8)	68	7.7 (1.9)	19	7.41 (1.4)	0.3 ± 0.5
CCI-all vs. usual care		−0.0 ± 0.2		−0.2 (0.3)		0.45 ± 0.43	
**C-Peptide (nmol L**^**−1**^**)**							
CCI-all education	248	4.36 (2.15)	185	4.40 (2.15)	63	4.25 (2.17)	0.15 ± 0.31
Usual Care	79	4.18 (2.48)	62	3.86 (2.22)	17	5.35 (3.08)	−1.50 ± 0.80
CCI-all vs. usual care		0.18 ± 0.29		0.54 ± 0.32		−1.10 ± 0.80	
**Fasting glucose (mg/dL)**							
CCI-all education	258	160.77 (61.37)	191	158.01 (60.77)	67	168.64 (62.86)	−10.63 ± 8.81
Usual Care	86	156.20 (72.60)	67	162.07 (78.71)	19	135.47 (39.85)	26.60 ± 13.27
CCI-all vs. usual care		4.57 ± 8.01		−4.06 ± 10.57		33.17 ± 15.25	
**Fasting Insulin (mIU L**^**−1**^**)**							
CCI-all education	248	28.56 (23.88)	185	27.37 (22.33)	63	32.06 (27.86)	−4.70 ± 3.87
Usual Care	79	29.11 (24.85)	62	25.54 (21.87)	17	42.12 (30.95)	−16.58 ± 6.58
CCI-all vs. usual care		−0.55 ± 3.12		1.83 ± 3.26		−10.05 ± 7.79	
**HOMA-IR (insulin derived), all**							
CCI-all education	220	8.96 (6.17)	168	8.92 (6.19)	52	9.10 (6.14)	−0.19 ± 0.98
Usual Care	78	10.64 (9.12)	61	9.56 (8.35)	17	14.52 (10.88)	−4.96 ± 2.85
CCI-all vs. usual care		−1.68 ± 1.11		−0.65 ± 1.17		−5.41 ± 2.77	
**HOMA-IR (insulin derived), excluding exogenous users**							
CCI-all education	157	8.80 (5.64)	121	8.62 (5.74)	36	9.41 (5.31)	−0.78 ± 1.07
Usual Care	42	9.41 (8.35)	32	7.95 (6.53)	10	14.09 (11.77)	−6.15 ± 2.90
CCI-all vs. usual care		−0.61 ± 1.36		0.68 ± 1.17		−4.68 ± 3.82	
**HOMA-IR (C-peptide derived), all**							
CCI-all education	244	11.73 (7.40)	182	11.52 (6.55)	62	12.33 (9.51)	−0.80 ± 1.09
Usual Care	78	11.10 (7.56)	61	10.63 (7.64)	17	12.80 (7.23)	−2.17 ± 2.07
CCI-all vs. usual care		0.62 ± 0.97		0.89 ± 1.01		−0.47 ± 2.49	
**METABOLIC AND BODY COMPOSITION**							
**Diabetes reversal (%)**^**a**^							
CCI-all education	262	12.2 ± 2.0	194	12.9 ± 2.4	68	10.3 ± 3.7	2.6 ± 4.6
Usual Care	87	20.7 ± 4.4	68	19.1 ± 4.8	19	26.3 ± 10.4	−7.2 ± 10.6
CCI-all vs. usual care		−8.5 ± 4.8		−6.2 ± 5.4		−16.0 ± 11.0	
**Metabolic syndrome (%)**							
CCI-all education	262	88.6 ± 2.0	194	88.7 ± 2.3	68	88.2 ± 4.0	0.4 ± 4.5
Usual Care	81	91.4 ± 3.1	62	93.6 ± 3.2	19	84.2 ± 9.0	9.3 ± 9.2
CCI-all vs. usual care		−2.8 ± 4.0		−4.9 ± 3.9		4.0 ± 8.7	
**Weight-clinic (kgs)**							
CCI-all education	257	116.50 (25.94)	190	115.97 (24.94)	67	117.98 (28.72)	−2.00 ± 3.69
Usual Care	83	105.63 (22.14)	64	105.32 (21.81)	19	106.67 (23.82)	−1.35 ± 5.82
CCI-all vs. usual care		10.87 ± 3.17^*^		10.65 ± 3.50		11.32 ± 7.21	
**Spine bone mineral density (g/cm**^**2**^**)**							
CCI-all education	238	1.20 (0.16)	178	1.20 (0.15)	60	1.21 (0.18)	−0.01 ± 0.03
**Central abdominal fat (kg)**							
CCI-all education	237	5.77 (1.69)	177	5.72 (1.69)	60	5.94 (1.72)	−0.22 ± 0.25
**Android: gynoid ratio**							
CCI-all education	238	1.27 (0.33)	178	1.26 (0.33)	60	1.31 (0.34)	−0.06 ± 0.05
**Lower extremity lean mass (kg)**							
CCI-all education	238	18.45 (4.05)	178	18.42 (3.94)	60	18.53 (4.40)	−0.11 ± 0.61
**CARDIOVASCULAR**							
**Systolic blood pressure (mmHg)**							
CCI-all education	260	131.9 (14.1)	192	132.2 (14.2)	68	131.1 (13.8)	1.2 (2.0)
Usual Care	79	129.8 (13.6)	61	129.0 (13.6)	18	132.7 (13.5)	−3.7 (3.7)
CCI-all vs. usual care		2.1 ± 1.8		3.3 ± 2.1		−1.6 ± 3.6	
**Diastolic blood pressure (mmHg)**							
CCI-all education	260	82.1 (8.3)	192	81.7 (8.0)	68	83.4 (8.9)	−1.7 ± 1.2
Usual Care	79	82.0 (8.9)	61	82.1 (8.8)	18	81.8 (9.6)	0.3 ± 2.4
CCI-all vs. usual care		0.1 ± 1.1		−0.4 ± 1.2		1.6 ± 2.4	
**Total cholesterol (mg/dL)**							
CCI-all education	247	183.6 (41.2)	184	181.9 (40.3)	63	188.7 (43.6)	−6.8 ± 6.0
Usual Care	79	183.8 (45.8)	62	186.5 (49.3)	17	174.0 (28.7)	12.5 ± 12.5
CCI-all vs. usual care		−0.2 ± 5.5		−4.6 ± 6.3		14.7 ± 11.2	
**LDL-cholesterol (mg/dL)**							
CCI-all education	232	102.5 (32.9)	173	101.1 (33.0)	59	106.6 (32.6)	−5.5 ± 5.0
Usual Care	70	101.5 (36.2)	56	103.8 (38.3)	14	92.3 (24.8)	11.5 ± 10.8
CCI-all vs. usual care		1.0 ± 4.6		−2.7 ± 5.3		14.3 ± 9.3	
**HDL-cholesterol (mg/dL)**							
CCI-all education	247	42.2 (13.4)	184	42.5 (13.7)	63	41.3 (12.7)	1.1 ± 2.0
Usual Care	79	37.6 (11.2)	62	38.3 (11.5)	17	35.2 (10.1)	3.0 ± 3.1
CCI-all vs. usual care		4.6 ± 1.7		4.2 ± 1.9		6.1 ± 3.3	
**Triglycerides (mg/dL)**							
CCI-all education	247	197.2 (143.4)	184	200.7 (153.5)	63	187.1 (109.0)	13.5 ± 21.0
Usual Care	79	282.9 (401.2)	62	283.7 (443.6)	17	280.0 (185.0)	3.7 ± 110.5
CCI-all vs. usual care		−85.7 ± 46.1		−83.0 ± 57.5		−92.9 ± 46.9	
**LIVER**							
**ALT (Units/L)**							
CCI-all education	257	30.65 (22.77)	190	31.65 (24.54)	67	27.79 (16.63)	3.86 ± 3.23
Usual Care	86	27.74 (19.81)	67	28.31 (21.30)	19	25.74 (13.59)	2.58 ± 5.17
CCI-all vs. usual care		2.90 ± 2.75		3.34 ± 3.38		2.05 ± 4.17	
**AST (Units/L)**							
CCI-all education	257	23.69 (15.19)	190	24.37 (16.79)	67	21.76 (9.08)	2.61 ± 2.16
Usual Care	86	23.90 (19.39)	67	24.25 (21.36)	19	22.63 (10.02)	1.62 ± 5.07
CCI-all vs. usual care		−0.20 ± 2.04		0.12 ± 2.57		−0.87 ± 2.42	
**ALP (Units/L)**							
CCI-all education	256	74.11 (22.14)	189	74.32 (22.32)	67	73.54 (21.79)	0.78 ± 3.15
Usual Care	86	77.36 (26.29)	67	78.25 (27.67)	19	74.21 (21.08)	4.04 ± 6.86
CCI-all vs. usual care		−3.25 ± 2.90		−3.94 ± 3.39		−0.67 ± 5.62	
**Bilirubin (mg/dL)**							
CCI-all education	256	0.54 (0.21)	189	0.55 (0.21)	67	0.49 (0.18)	0.06 ± 0.03
Usual Care	86	0.55 (0.28)	67	0.54 (0.27)	19	0.59 (0.29)	−0.05 ± 0.07
CCI-all vs. usual care		−0.02 ± 0.03		0.01 ± 0.04		−0.11 ± 0.05	
**NAFLD-Liver fat score**							
CCI-all education	243	3.43 (3.84)	181	3.26 (3.62)	62	3.92 (4.44)	−0.65 ± 0.62
Usual Care	74	3.10 (3.63)	57	2.49 (3.00)	17	5.14 (4.80)	−2.65 ± 1.23
CCI-all vs. usual care		0.33 ± 0.50		0.78 ± 0.53		−1.23 ± 1.24	
**NAFLD-Fibrosis score**							
CCI-all education	238	−0.23 (1.36)	177	−0.25 (1.37)	61	−0.18 (1.35)	−0.07 ± 0.20
Usual Care	75	−0.80 (1.41)	58	−0.82 (1.47)	17	−0.71 (1.20)	−0.11 ± 0.39
CCI-all vs. usual care		0.56 ± 0.18		0.57 ± 0.21		0.53 ± 0.36	
**KIDNEY**							
**Anion gap (mmol L**^**−1**^**)**							
CCI-all education	257	6.83 (1.67)	190	6.76 (1.68)	67	7.03 (1.62)	−0.27 ± 0.24
Usual Care	86	6.93 (1.82)	67	6.82 (1.86)	19	7.32 (1.67)	−0.50 ± 0.47
CCI-all vs. usual care		−0.10 ± 0.21		−0.06 ± 0.25		−0.29 ± 0.42	
**BUN (mg/dL)**							
CCI-all education	258	16.88 (6.55)	191	17.17 (6.05)	67	16.06 (7.81)	1.11 ± 0.93
Usual Care	86	16.05 (6.25)	67	15.81 (6.28)	19	16.89 (6.24)	−1.09 ± 1.63
CCI-all vs. usual care		0.84 ±−0.81		1.37 ± 0.87		−0.84 ± 1.95	
**eGFR (mL s**^**−1**^ **m**^**−2**^**)**							
CCI-all education	258	80.48 (13.62)	191	80.36 (13.53)	67	80.84 (13.96)	−0.48 ± 1.94
Usual Care	86	79.17 (13.73)	67	79.39 (13.72)	19	78.42 (14.11)	0.97 ± 3.59
CCI-all vs. usual care		1.31 ± 1.70		0.97 ± 1.93		2.42 ± 3.64	
**Serum creatinine (mg/dL)**							
CCI-all education	258	0.88 (0.24)	191	0.88 (0.23)	67	0.90 (0.26)	−0.02 ± 0.03
Usual Care	86	0.91 (0.25)	67	0.91 (0.25)	19	0.90 (0.22)	0.004 ± 0.06
CCI-all vs. usual care		−0.02 ± 0.03		−0.03 ± 0.03		−0.01 ± 0.07	
**Uric acid (mg/dL)**							
CCI-all education	261	5.85 (1.46)	193	5.88 (1.45)	68	5.77 (1.48)	0.11 ± 0.21
Usual Care	85	5.60 (1.47)	67	5.58 (1.34)	18	5.70 (1.92)	0.12 ± 0.39
CCI-all vs. usual care		0.25 ± 0.18		0.30 ± 0.20		0.07 ± 0.42	
**THYROID**							
**TSH (mIU L**^**−1**^**)**							
CCI-all education	259	2.32 (1.74)	192	2.31 (1.81)	67	2.36 (1.52)	−0.05 ± 0.25
Usual Care	86	3.80 (17.07)	68	4.37 (19.17)	18	1.65 (1.05)	2.72 ± 4.54
CCI-all vs. usual care		−1.48 ± 1.84		−2.06 ± 2.33		0.71 ± 0.38	
**Free T4 (ng/dL)**							
CCI-all education	260	0.92 (0.17)	193	0.92 (0.18)	67	0.91 (0.17)	0.01 ± 0.02
Usual Care	86	0.88 (0.29)	68	0.87 (0.31)	18	0.89 (0.16)	−0.02 ± 0.08
CCI-all vs. usual care		0.04 ± 0.03		0.05 ± 0.03		0.02 ± 0.04	
**OTHER**							
**Beta-hydroxybutyrate (mmol L**^**−1**^**)**							
CCI-all education	248	0.17 (0.15)	185	0.17 (0.15)	63	0.19 (0.16)	−0.03 ± 0.02
Usual Care	79	0.15 (0.13)	62	0.14 (0.11)	17	0.20 (0.18)	−0.06 ± 0.04
CCI-all vs. usual care		0.02 ± 0.20		0.03 ± 0.18		−0.01 (0.04)	
**hsC-reactive protein (nmol L**^**−1**^**)**							
CCI-all education	249	8.54 (14.49)	186	8.92 (16.35)	63	7.44 (6.41)	1.48 ± 2.12
Usual Care	85	8.89 (8.62)	67	9.08 (8.91)	18	8.18 (7.64)	0.90 ± 2.30
CCI-all vs. usual care		−0.34 ± 1.67		−0.16 ± 2.10		−0.74 ± 1.79	
**White blood cell (k/cumm)**							
CCI-all education	260	7.24 (1.89)	193	7.12 (1.82)	67	7.57 (2.08)	−0.45 ± 0.27
Usual Care	86	8.14 (2.39)	67	8.15 (2.30)	19	8.08 (2.73)	0.07 ± 0.62
CCI-all vs. usual care		−0.90 ± 0.28		−1.03 ± 0.31^*^		−0.51 ± 0.58	
**DIABETES MEDICATION**							
**Any diabetes medication, excluding metformin (%)**							
CCI-all education	262	56.87 ± 3.07	194	55.67 ± 3.58	68	60.29 ± 5.98	−4.62 ± 7.00
Usual Care	87	66.67 ± 5.08	68	66.18 ± 5.78	19	68.42 ± 10.96	−2.25 ± 12.37
CCI-all vs. usual care		−9.80 ± 5.94		−10.51 ± 6.80		−8.13 ± 12.71	
**Sulfonylurea (%)**							
CCI-all education	262	23.66 ± 2.63	194	25.77 ± 3.15	68	17.65 ± 4.66	8.13 ± 5.62
Usual Care	87	24.14 ± 4.61	68	22.06 ± 5.07	19	31.58 ± 10.96	−9.52 ± 11.19
CCI-all vs. usual care		−0.47 ± 5.28		3.71 ± 6.11		−13.93 ± 11.91	
**Insulin (%)**							
CCI-all education	262	29.77 ± 2.83	194	29.38 ± 3.28	68	30.88 ± 5.64	−1.50 ± 6.47
Usual Care	87	45.98 ± 5.37	68	48.53 ± 6.11	19	36.84 ± 11.37	11.69 ± 12.91
CCI-all vs. usual care		−16.21 ± 6.07		−19.15 ± 6.93		−5.96 ± 12.25	
**Thiazolidinedione (%)**							
CCI-all education	262	1.53 ± 0.76	194	1.55 ± 0.89	68	1.47 ± 01.47	0.08 ± 1.74
Usual Care	87	1.15 ± 1.15	68	1.47 ± 1.47	19	0.00 ± 0.00	1.47 ± 2.79
CCI-all vs. usual care		0.38 ± 1.48		0.08 ± 1.74		1.47 ± 2.79	
**SGLT-2 (%)**							
CCI-all education	262	10.31 ± 1.88	194	9.79 ± 2.14	68	11.77 ± 3.94	−1.97 ± 4.30
Usual Care	87	14.94 ± 3.84	68	14.71 ± 4.33	19	15.79 ± 8.59	−1.08 ± 9.36
CCI-all vs. usual care		−4.64 ± 4.28		−4.91 ± 4.83		−4.03 ± 8.71	
**DPP-4 (%)**							
CCI-all education	262	9.92 ± 1.85	194	9.28 ± 2.09	68	11.77 ± 3.94	−2.49 ± 4.23
Usual Care	87	8.05 ± 2.93	68	5.88 ± 2.87	19	15.79 ± 8.59	−9.91 ± 9.06
CCI-all vs. usual care		1.88 ± 3.63		3.40 ± 3.92		−4.03 ± 8.71	
**GLP-1 (%)**							
CCI-all education	262	13.36 ± 2.11	194	13.40 ± 2.45	68	13.24 ± 4.14	0.17 ± 4.81
Usual Care	87	16.09 ± 3.96	68	19.12 ± 4.80	19	5.26 ± 5.26	13.85 ± 7.13
CCI-all vs. usual care		−2.73 ± 4.31		−5.72 ± 5.39		7.97 ± 8.33	
**Metformin (%)**							
CCI-all education	262	71.37 ± 2.80	194	71.65 ± 3.24	68	70.59 ± 05.57	1.06 ± 6.39
Usual Care	87	60.92 ± 5.26	68	60.29 ± 5.98	19	63.16 ± 11.37	−2.86 ± 12.81
CCI-all vs. usual care		10.46 ± 5.96		11.36 ± 6.80		7.43 ± 12.12	

SD, standard deviation; SE, standard error; CCI, continuous care intervention; UC, usual care; HOMA-IR, homeostatic model assessment of insulin resistance; LDL, low-density lipoprotein; HDL, high-density lipoprotein; ALT, alanine aminotransferase; AST, aspartate aminotransferase; ALP, alkaline phosphatase; NAFLD, nonalcoholic fatty liver disease; BUN, blood urea nitrogen; eGFR, estimated glomerular filtration rates; TSH, thyroid stimulating hormone; SGLT-2, Sodium glucose co-transporter 2 inhibitor; DPP-4, Dipeptidyl peptidase-4 inhibitor; GLP-1, Glucagon-like peptide 1 receptor agonist.

*Ns slightly differs for each variable depending on the patients' compliance to complete their laboratory, body composition assessments and clinic visits within the specified time-frame*.

a Meeting diabetes reversal criteria at baseline was defined as HbA1c <6.5% and no use of medication for glycemic control other than metformin.

* *A significance level of P < 0.0012 ensures overall simultaneous significance of P < 0.05 over the 43 variables using Bonferroni correction*.

### Retention and Long-Term Dietary Adherence

One hundred ninety four participants (74% of 262) remained enrolled in the CCI at 2 years (Figure 1), as did 68 UC group participants (78% of 87). CCI-participant-reported reasons for dropout included: intervening life events (e.g., family emergencies), difficulty attending or completing laboratory and clinic visits associated with the trial, and insufficient motivation for participation in the intervention. At both 1 and 2 years, laboratory-measured blood BHB was 0.27 ± 0.02 mmol L^−1^, 50% higher than the baseline value (0.18 ± 0.01 mmol L^−1^). The mean laboratory BHB level was stable from 1 to 2 years, and 61.5% (*n* = 161) of participants uploaded a blood BHB measurement >0.5 mmol L^−1^ in the app at least once between 1 and 2 years.

### Glycemic Control

**Figure 2:**
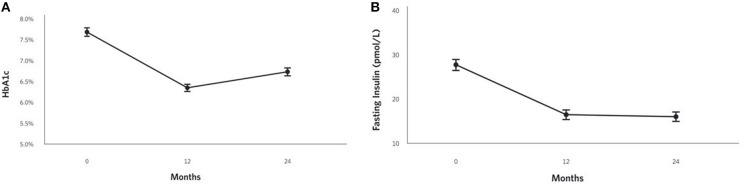
Adjusted mean changes from baseline to 2 years in the CCI group for **(A)** HbA1c (−12% relative to baseline, *P* = 1.8 × 10^−17^), **(B)** Fasting insulin (−42% relative to baseline, *P* = 2.2 × 10^−18^).

HbA1c improved at 2 years (0.9% unit decrease, *P* = 1.8 × 10^−17^; Figure 2A) among CCI participants and was lower than the UC group. Related markers including C-peptide, fasting glucose, fasting insulin (Figure 2B), insulin-derived HOMA-IR excluding exogenous insulin users, and c-peptide-derived HOMA-IR also significantly decreased after correction for multiple comparisons in the CCI group at 2 years and were lower than the UC group (except C-peptide); no changes from baseline to 2 years were observed in the UC group (Supplementary Figures 1A,B; Table 2).

**Table 2:** Adjusted mean changes over time.

	**Baseline**		**1 Year**				**2 Years**			
	**Mean ± SE**	**P**	**Mean ± SE**	***P***	**Change from baseline**	***P***	**Mean ± SE**	***P***	**Change from baseline**	***P***
**GLYCEMIC**										
**Hemoglobin A1c (%)**										
CCI-all education	7.7 ± 0.1		6.3 ± 0.1		−1.3 ± 0.1	6.6 × 10^−38^	6.7 ± 0.1		−0.9 ± 0.1	1.8 × 10^−17^
Usual Care	7.5 ± 0.2		7.6 ± 0.1		0.2 ± 0.2	0.31	7.9 ± 0.2		0.4 ± 0.2	0.02
CCI-all vs. usual care	0.2 ± 0.2	0.28	−1.3 ± 0.2	2.7 × 10^−14^			−1.2 ± 0.2	1.3 × 10^−9^		
**C-Peptide (nmol L**^**−1**^**)**										
CCI-all education	4.33 ± 0.13		3.27 ± 0.14		−1.06 ± 0.13	7.3 × 10^−14^	3.16 ± 0.12		−1.17 ± 0.13	2.2 × 10^−16^
Usual Care	4.39 ± 0.24		4.38 ± 0.25		−0.004 ± 0.24	0.99	3.89 ± 0.22		−0.49 ± 0.24	0.04
CCI-all vs. usual care	−0.06 ± 0.28	0.84	−1.12 ± 0.28	9.8 × 10^−5^			−0.73 ± 0.26	5.0 × 10^−3^		
**Fasting glucose (mg/dL)**										
CCI-all education	163.67 ± 3.90		127.29 ± 3.62		−36.39 ± 4.47	1.0 × 10^−14^	134.58 ± 4.13		−29.10 ± 4.88	6.8 × 10^−9^
Usual Care	151.21 ± 6.93		160.58 ± 6.17		9.38 ± 7.61	0.22	172.89 ± 7.00		21.68 ± 8.28	0.01
CCI-all vs. usual care	12.47 ± 8.02	0.12	−33.30 ± 7.24	6.3 × 10^−6^			−38.31 ± 8.21	4.8 × 10^−6^		
**Fasting Insulin (mIU L**^**−1**^**)**^**a**^										
CCI-all education	27.73 ± 1.26		16.47 ± 1.13		−11.26 ± 1.28	3.2 × 10^−16^	16.02 ± 1.02		−11.71 ± 1.25	2.2 × 10^−18^
Usual Care	27.57 ± 2.29		26.47 ± 2.06		−1.10 ± 2.30	0.63	24.17 ± 1.84		−3.40 ± 2.22	0.13
CCI-all vs. usual care	0.16 ± 2.63	0.95	−10.00 ± 2.38	3.6 × 10^−5^			−8.15 ± 2.14	1.7 × 10^−4^		
**HOMA-IR (insulin derived), all**^**a**^										
CCI-all education	9.09 ± 0.41		4.85 ± 0.39		−4.24 ± 0.45	3.5 × 10^−18^	5.27 ± 0.44		−3.82 ± 0.49	3.8 × 10^−13^
Usual Care	9.58 ± 0.73		10.33 ± 0.73		0.75 ± 0.81	0.35	9.95 ± 0.77		0.37 ± 0.83	0.66
CCI-all vs. usual care	−0.49 ± 0.85	0.57	−5.48 ± 0.84	2.9 × 10^−10^			−4.67 ± 0.89	3.4 × 10^−7^		
**HOMA-IR (insulin derived), excluding exogenous users**^**a**^										
CCI-all education	9.08 ± 0.46		4.56 ± 0.44		−4.53 ± 0.47	6.5 × 10^−18^	5.25 ± 0.38		−3.83 ± 0.49	2.7 × 10^−13^
Usual Care	8.66 ± 0.92		10.87 ± 0.98		2.21 ± 1.02	0.03	8.26 ± 0.75		−0.40 ± 0.94	0.68
CCI-all vs. usual care	0.43 ± 1.03		−6.31 ± 1.08	2.2 × 10^−8^			−3.01 ± 0.85	5.4 × 10^−4^		
**HOMA-IR (C-peptide derived), all**^**a**^										
CCI-all education	11.25 ± 0.37		8.07 ± 0.38		−3.19 ± 0.39	1.8 × 10^−14^	7.88 ± 0.35		−3.37 ± 0.39	1.1 × 10^−15^
Usual Care	11.04 ± 0.67		11.81 ± 0.71		0.77 ± 0.72	0.28	10.62 ± 0.64	2.5 × 10^−4^	−0.42 ± 0.70	0.55
CCI-all vs. usual care	0.21 ± 0.77	0.78	−3.75 ± 0.81	5.8 × 10^−6^			−2.74 ± 0.74			
**METABOLIC AND BODY COMPOSITION**										
**Weight-clinic (kg)**										
CCI-all education	114.56 ± 0.60		100.27 ± 0.86		−14.29 ± 0.71	9.7 × 10^−56^	102.62 ± 1.10		−11.94 ± 0.96	8.8 × 10^−28^
Usual Care	111.07 ± 1.09		111.71 ± 1.47		0.64 ± 1.17	0.58	112.35 ± 1.90		1.28 ± 1.63	0.43
CCI-all vs. usual care	3.49 ± 1.27	0.01	−11.44 ± 1.71	1.4 × 10^−10^			−9.73 ± 2.20	1.5 × 10^−5^		
**Spine bone mineral density (g/cm**^**2**^**)**										
CCI-all education	1.21 ± 0.01	—	1.22 ± 0.01	—	0.01 ± 0.01	0.11	1.22 ± 0.01	—	0.01 ± 0.01	0.02
**Central abdominal fat (kg)**										
CCI-all education	5.89 ± 0.07	—	4.62 ± 0.08	—	−1.27 ± 0.07	1.3 × 10^−42^	4.99 ± 0.10	—	−0.90 ± 0.08	1.6 × 10^−21^
**Android: gynoid ratio**										
CCI-all education	1.27 ± 0.02	—	1.18 ± 0.02	—	−0.09 ± 0.1	2.4 × 10^−13^	1.20 ± 0.02	—	−0.07 ± 0.01	4.7 × 10^−8^
**Lower extremities lean mass (kg)**										
CCI-all education	18.74 ± 0.16	—	17.41 ± 0.15	—	−1.33 ± 0.10	5.9 × 10^−31^	17.38 ± 0.17	—	−1.36 ± 0.12	1.3 × 10^−21^
**CARDIOVASCULAR**										
**Systolic blood pressure (mmHg)**										
CCI-all education	131.7 ± 0.9		125.3 ± 0.9		−6.5 ± 1.1	3.3 × 10^−8^	125.9 ± 1.0		−5.8 ± 1.2	2.4 × 10^−6^
Usual Care	130.3 ± 1.6		129.5 ± 1.6		−0.9 ± 1.9	0.66	129.9 ± 1.8		−0.5 ± 2.1	0.83
CCI-all vs. usual care	1.4 ± 1.8	0.43	−4.2 ± 1.8	0.02			−3.9 ± 2.1	0.06		
**Diastolic blood pressure (mmHg)**										
CCI-all education	81.8 ± 0.5		78.1 ± 0.6		−3.7 ± 0.7	5.4 × 10^−8^	78.7 ± 0.6		−3.1 ± 0.7	3.3 × 10^−5^
Usual Care	82.1 ± 1.0		81.3 ± 1.0		−0.8 ± 1.1	0.47	81.6 ± 1.1		−0.6 ± 1.3	0.65
CCI-all vs. usual care	−0.3 ± 1.1	0.76	−3.2 ± 1.1	0.41			−2.8 ± 1.3	0.03		
**Total cholesterol (mg/dL)**										
CCI-all education	184.4 ± 2.7		192.8 ± 3.4		8.4 ± 3.1	0.01	194.1 ± 3.5		9.7 ± 3.6	0.01
Usual Care	181.2 ± 4.9		179.4 ± 6.1		−1.8 ± 5.5	0.75	180.9 ± 6.2		−0.3 ± 6.4	0.96
CCI-all vs. usual care	3.3 ± 5.7	0.57	13.5 ± 7.0	0.06			13.3 ± 7.2	0.07		
**LDL-cholesterol (mg/dL)**										
CCI-all education	103.5 ± 2.2		114.1 ± 2.5		10.6 ± 2.5	2.5 × 10^−5^	114.6 ± 2.8		11.1 ± 2.8	1.1x 10^−4^
Usual Care	100.0 ± 4.2		88.9 ± 4.9		−11.2 ± 4.7	0.02	90.9 ± 5.1		−9.1 ± 5.1	0.08
CCI-all vs. usual care	3.6 ± 4.8	0.46	25.2 ± 5.6	8.9 × 10^−6^			23.7 ± 5.9	7.0x 10^−5^		
**HDL-cholesterol (mg/dL)**										
CCI-all education	41.8 ± 0.9		49.5 ± 0.9		7.8 ± 0.8	4.4 × 10^−19^	49.5 ± 1.0		7.8 ± 0.9	2.7 × 10^−16^
Usual Care	38.7 ± 1.4		37.2 ± 1.7		−1.5 ± 1.4	0.3	42.5 ± 1.7		3.8 ± 1.6	0.02
CCI-all vs. usual care	3.1 ± 1.6	0.06	12.4 ± 2.0	1.1 × 10^−9^			7.1 ± 2.0	4.1x 10^−4^		
**Triglycerides (mg/dL)**^**b**^										
CCI-all education	197.2 ± 9.1		148.9 ± 10.1		−48.3 ± 13.7	7.4 × 10^−16^	153.3 ± 10.4		−43.9 ± 14.0	6.2 × 10^−9^
Usual Care	282.9 ± 45.1		314.5 ± 61.4		31.6 ± 74.6	0.35	209.5 ± 18.5		−73.4 ± 55.9	0.75
CCI-all vs. usual care	−85.7 ± 30.1	0.09	−165.5 ± 39.0	1.5 × 10^−8^			−56.2 ± 19.0	7.1 × 10^−5^		
**LIVER**										
**ALT (Units/L)**^**a**^										
CCI-all education	29.16 ± 0.97		21.53 ± 0.88		−7.63 ± 1.02	7.7 × 10^−13^	23.00 ± 0.91		−6.16 ± 0.95	4.0 × 10^−10^
Usual Care	25.84 ± 1.72		26.98 ± 1.51		1.14 ± 1.73	0.51	26.80 ± 1.57		0.96 ± 1.62	0.56
CCI-all vs. usual care	3.31 ± 1.99	0.1	−5.45 ± 1.77	0.002			−3.80 ± 1.84	0.04		
**AST (Units/L)**^**a**^										
CCI-all education	22.50 ± 0.64		19.07 ± 0.58		−3.43 ± 0.69	1.1 × 10^−6^	19.78 ± 0.57		−2.72 ± 0.66	5.1 × 10^−5^
Usual Care	21.51 ± 1.13		23.37 ± 1.00		1.86 ± 1.19	0.12	23.19 ± 0.99		1.68 ± 1.14	0.14
CCI-all vs. usual care	0.99 ± 1.31	0.45	−4.30 ± 1.17	2.8 × 10^−4^			−3.41 ± 1.16	3.5 × 10^−3^		
**ALP (Units/L)**										
CCI-all education	74.13 ± 1.42		64.34 ± 1.44		−9.78 ± 0.98	1.9 × 10^−20^	64.50 ± 1.58		−9.63 ± 1.19*	1.8 × 10^−14^
Usual Care	78.55 ± 2.53		79.05 ± 2.55		0.50 ± 1.65	0.76	82.47 ± 2.76		3.92 ± 2.00	0.05
CCI-all vs. usual care	−4.42 ± 2.94	0.13	−14.71 ± 2.97	1.2 × 10^−6^			−17.97 ± 3.22	5.1 × 10^−8^		
**Bilirubin (mg/dL)**^**a**^										
CCI-all education	0.53 ± 0.01		0.53 ± 0.02		−0.001 ± 0.01	0.92	0.52 ± 0.02		−0.01 ± 0.01	0.45
Usual Care	0.55 ± 0.02		0.57 ± 0.03		0.03 ± 0.02	0.16	0.52 ± 0.03		−0.03 ± 0.02	15
CCI-all vs. usual care	−0.01 ± 0.03	0.64	−0.04 ± 0.03	0.18			0.01 ± 0.03	0.8		
**NAFLD-Liver fat score**^**a**^										
CCI-all education	3.29 ± 0.21		1.34 ± 0.19		−1.95 ± 0.22	2.0 × 10^−16^	0.71 ± 0.20		−2.58 ± 0.22	2.9 × 10^−25^
Usual Care	3.20 ± 0.38		3.79 ± 0.35		0.59 ± 0.40	0.14	3.02 ± 0.37		−0.17 ± 0.40	0.66
CCI-all vs. usual care	0.09 ± 0.44	0.83	−2.45 ± 0.40	4.2 × 10^−9^			−2.32 ± 0.43	1.6 × 10^−7^		
**NAFLD-Fibrosis score**										
CCI-all education	−0.31 ± 0.06		−0.95 ± 0.07		−0.64 ± 0.06	4.0 × 10^−22^	−0.78 ± 0.08		−0.47 ± 0.08	2.3 × 10^−9^
Usual Care	−0.45 ± 0.11		−0.19 ± 0.12		0.27 ± 0.12	0.01	−0.24 ± 0.14		0.21 ± 0.14	0.12
CCI-all vs. usual care	0.14 ± 0.13	0.27	−0.77 ± 0.14	4.4 × 10^−8^			−0.54 ± 0.16	0.001		
**KIDNEY**										
**Anion gap (mmol L**^**−1**^**)**										
CCI-all education	6.83 ± 0.11		7.12 ± 0.13		0.29 ± 0.15	0.05	7.29 ± 0.13		0.46 ± 0.14	0.003
Usual Care	6.92 ± 0.19		7.74 ± 0.22		0.82 ± 0.25	0.001	7.80 ± 0.22		0.88 ± 0.24	3.2 × 10^−4^
CCI-all vs. usual care	−0.09 ± 0.22	0.68	−0.63 ± 0.25	0.01			−0.51 ± 0.25	0.04		
**BUN (mmol L**^**−1**^**)**^**a**^										
CCI-all education	16.40 ± 0.32		18.46 ± 0.37		2.06 ± 0.36	3.8 × 10^−8^	17.41 ± 0.40		1.01 ± 0.43	0.02
Usual Care	16.18 ± 0.56		15.83 ± 0.63		−0.35 ± 0.61	0.57	16.21 ± 0.68		0.03 ± 0.72	0.97
CCI-all vs. usual care	0.22 ± 0.65	0.74	2.63 ± 0.74	4.0 × 10^−4^			1.20 ± 0.90	0.14		
**eGFR (mL s**^**−1**^ **m**^**−2**^**)**										
CCI-all education	80.53 ± 0.78		82.50 ± 0.78		1.97 ± 0.67	0.004	83.26 ± 0.80		2.73 ± 0.72	1.6 × 10^−4^
Usual Care	78.70 ± 1.39		79.56 ± 1.36		0.86 ± 1.13	0.45	79.12 ± 1.39		0.42 ± 1.21	0.73
CCI-all vs. usual care	1.82 ± 1.61	0.26	2.94 ± 1.59	0.07			4.14 ± 1.63	0.01		
**Serum creatinine (μmol L**^**−1**^**)**^**a**^										
CCI-all education	0.88 ± 0.01		0.83 ± 0.01		−0.04 ± 0.01	5.3 × 10^−6^	0.85 ± 0.01		−0.03 ± 0.01	0.003
Usual Care	0.90 ± 0.02		0.87 ± 0.02		−0.03 ± 0.02	0.07	0.88 ± 0.02		−0.01 ± 0.02	0.39
CCI-all vs. usual care	−0.02 ± 0.02	0.37	−0.04 ± 0.02	0.12			−0.04 ± 0.02	0.12		
**Uric acid (μmo L**^**−1**^**)**										
CCI-all education	5.83 ± 0.09		5.82 ± 0.10		−0.01 ± 0.08	0.9	5.72 ± 0.10		−0.11 ± 0.09	0.2
Usual Care	5.67 ± 0.16		5.44 ± 0.18		−0.24 ± 0.14	0.09	5.13 ± 0.18		−0.54 ± 0.16	6.2 × 10^−4^
CCI-all vs. usual care	0.16 ± 0.19	0.39	0.39 ± 0.21	0.06			0.59 ± 0.21	0.005		
**THYROID**										
**TSH (mIU L**^**−1**^**)**^**a**^										
CCI-all education	2.16 ± 0.08		1.89 ± 0.07		−0.28 ± 0.07*	1.3 × 10^−4^	1.90 ± 0.08		−0.22 ± 0.09	0.01
Usual Care	1.94 ± 0.14		1.92 ± 0.13		−0.01 ± 0.12	0.92	2.04 ± 0.14		0.11 ± 0.16	0.49
CCI-all vs. usual care	0.23 ± 0.16	0.15	−0.04 ± 0.15	0.79			−0.10 ± 0.16	0.52		
**Free T4 (pmol L**^**−1**^**)**^**a**^										
CCI-all education	0.91 ± 0.01		0.92 ± 0.01		0.01 ± 0.01	0.04	0.93 ± 0.01		0.01 ± 0.01	0.01
Usual Care	0.85 ± 0.02		0.89 ± 0.02		0.04 ± 0.02	0.53	0.90 ± 0.02		0.05 ± 0.02	0.25
CCI-all vs. usual care	0.06 ± 0.02	0.003	0.03 ± 0.03	0.23			0.02 ± 0.03	0.34		
**Other**										
**Beta-hydroxybutyrate (mmol L**^**−1**^**)**^**a**^										
CCI-all education	0.18 ± 0.01		0.27 ± 0.02		0.09 ± 0.02	6.8 × 10^−7^	0.27 ± 0.02		0.09 ± 0.02	4.7 × 10^−5^
Usual Care	0.14 ± 0.02		0.17 ± 0.03		0.03 ± 0.03	0.43	0.18 ± 0.04		0.03 ± 0.04	0.38
CCI-all vs. usual care	0.03 ± 0.02	0.11	0.10 ± 0.04	0.01			0.09 ± 0.04	0.03		
**hsC-reactive protein (nmol L**^**−1**^**)**^**a**^										
CCI-all education	7.45 ± 0.42		5.01 ± 0.46		−2.44 ± 0.40	2.4 × 10^−9^	4.69 ± 0.40		−2.76 ± 0.37	6.9 × 10^−13^
Usual Care	9.03 ± 0.75		9.06 ± 0.81		0.03 ± 0.69	0.96	8.38 ± 0.74		−0.65 ± 0.65	0.32
CCI-all vs. usual care	−1.58 ± 0.87	0.07	−4.05 ± 0.94	2.1 × 10^−5^			−3.69 ± 0.86	2.3 × 10^−5^		
**White blood cell (k/cumm)**										
CCI-all education	7.22 ± 0.12		6.52 ± 0.13		−0.70 ± 0.10	6.6 × 10^−11^	6.68 ± 0.15		−0.54 ± 0.13	4.3 × 10^−5^
Usual Care	8.12 ± 0.22		8.16 ± 0.23		0.04 ± 0.17	0.82	8.07 ± 0.27		−0.05 ± 0.23	0.82
CCI-all vs. usual care	−0.90 ± 0.26	5.3 × 10^−4^	−1.64 ± 0.27*	2.3 × 10^−9^			−1.39 ± 0.32	1.6 × 10^−5^		

*Ns for continuous care intervention = 262 and Ns for usual care = 87. Unless otherwise noted, estimates reported were obtained from linear mixed-effects models which provide adjusted means and mean changes, controlling for baseline age, sex, race, body mass index, and insulin use. This maximum likelihood-based approach uses all available repeated data, resulting in an intent-to-treat analysis. A significance level of P < 0.0012 ensures overall simultaneous significance of P < 0.05 over the 43 variables using Bonferroni correction. Abbreviations: SE, standard error; CCI, continuous care intervention; UC, usual care; HOMA-IR, homeostatic model assessment of insulin resistance; LDL, low-density lipoprotein; HDL, high-density lipoprotein; ALT, alanine aminotransferase; AST, aspartate aminotransferase; ALP, alkaline phosphatase; NAFLD, nonalcoholic fatty liver disease; BUN, blood urea nitrogen; eGFR, estimated glomerular filtration rates; TSH, thyroid stimulating hormone*.

a Variable was positively skewed and after removing the top 1% of values, skew and kurtosis values fell within acceptable ranges. Analyses were conducted on data excluding the top 1% of values for each variable, although due to the maximum likelihood approach all cases were still included in the analyses.

b *Variable was positively skewed and a natural log transformation was performed. The linear mixed-effects model analysis including covariates was conducted on the transformed variable and significance values provided are from the transformed analysis. However, because transformed numbers are difficult to interpret, non-transformed and unadjusted means, mean changes, and standard errors for participants who completed the study visit were computed and provided in the table*.

**Figure 3:**
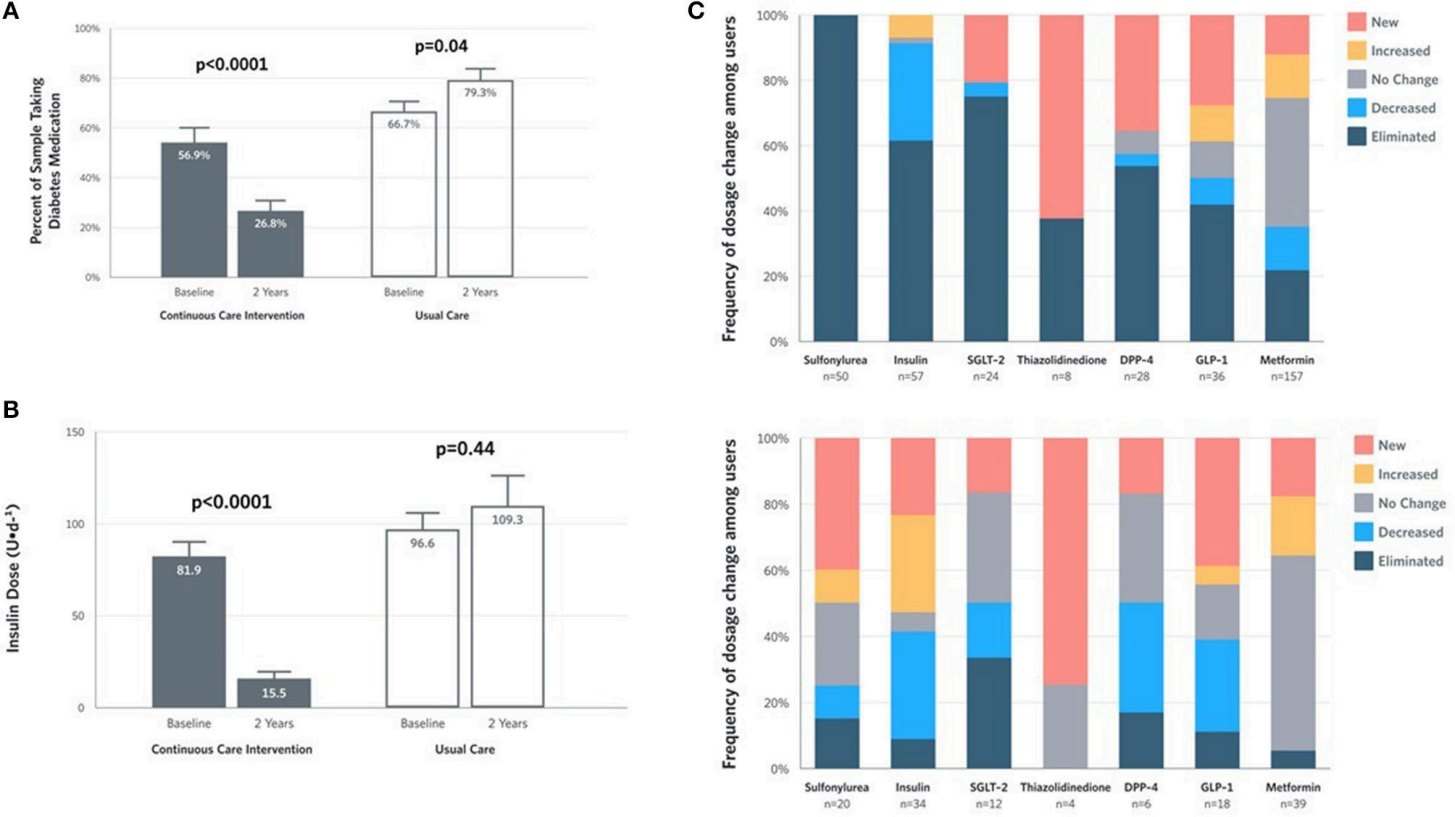
Medication and insulin dose changes from baseline to 2 years for CCI and UC group completers. **(A)** Percent of completers taking diabetes medications, excluding metformin. **(B)** Mean ± SE prescribed insulin dose among baseline users. **(C)** Frequency of medication dosage and use change among prescribed users by diabetes medication class.

Within the CCI, reduction in glycemia occurred concurrently with reduced medication use (Supplementary Table 3). The proportion of CCI completers taking any diabetes medication (excluding metformin) decreased at 2 years (Figure 3A). The mean dose among CCI participants prescribed insulin at baseline decreased by 81% at 2 years (from 81.9 to 15.5 U/day), but not among UC participants (+13%; from 96.6 to 109.3 U/day) (Figure 3B). For participants who remained insulin-users at 2 years, mean dose also decreased in the CCI by 61% (from 104.3 to 40.2 U/day, *P* = 9.2 × 10^−5^) but not in UC participants (+19% from 103.8 to 123.5 U/day, *P* = 0.29). Among completers prescribed each diabetes medication class, the proportion with each dosage change (eliminated, reduced, unchanged, increased or newly added) at 2 years in each group appears in Figure 3C.

### Diabetes Status

All within-group changes and between-group differences in diabetes status among the CCI and UC group participants appear in Supplementary Table 4 (intent-to-treat analyses were conducted, all below ns = 262). The proportion of participants meeting the defined criteria for diabetes reversal at 2 years increased to 53.5% from baseline in the CCI group, whereas no change was observed in the UC group. Diabetes remission (partial or complete) was observed in 46 (17.6%) participants in the CCI group and two (2.4%) of the UC participants at 2 years. Complete remission was observed in 17 (6.7%) CCI participants and none (0%) of the UC participants at 2 years.

### Weight and Body Composition Outcomes

**Figure 4:**
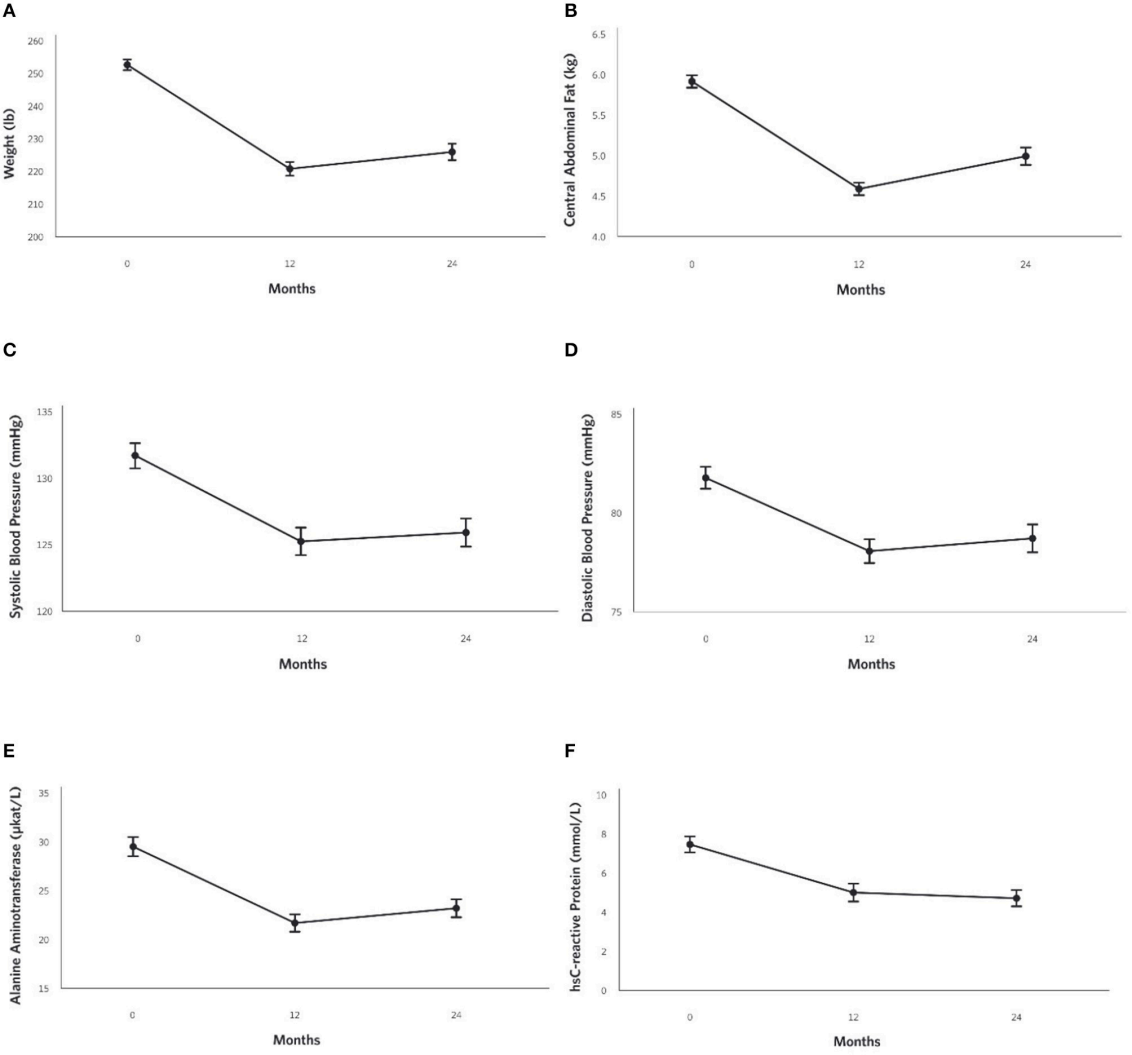
Adjusted mean changes from baseline to 2-years in the CCI group for **(A)** Weight (−10% relative to baseline, *P* = 8.8 × 10^−28^), **(B)** Central Abdominal Fat [CAF] (−15% relative to baseline, *P* = 1.6 × 10^−21^), **(C)** Systolic Blood Pressure (−4% relative to baseline, *P* = 2.4 × 10^−6^), **(D)** Diastolic Blood Pressure (−4% relative to baseline, *P* = 3.3 × 10^−5^) **(E)** Alanine aminotransferase [ALT] (−21% relative to baseline, *P* = 4.0 × 10^−10^), and **(F)** High sensitive C-reactive protein [hsCRP](−37% relative to baseline, *P* = 6.9 × 10^−13^).

At 2 years, the mean weight reduction from baseline was −10% (Figure 4A) in the CCI group, whereas no change was observed in the UC group (Supplementary Figure 1C). Among CCI patients, 74% had ≥5% weight loss compared to only 14% of UC patients (Supplementary Figure 2; completers analysis, *n* = 193). Consistent with the weight loss observed, the CCI group had reductions in abdominal fat content with decreases in CAF (Figure 4B) and the A/G ratio from baseline to 2 years (Table 2). Total spine BMD within the CCI remained unchanged from baseline to 2 years after correction for multiple comparisons, whereas the average LELM was reduced from baseline to 2 years (Table 2).

### Cardiovascular Risk Factor Outcomes

Decreases in systolic (Figure 4C) and diastolic (Figure 4D) blood pressures and triglycerides were observed in the CCI but not UC group at 2 years (Table 2; Supplementary Figures 3A,B). The CCI group's HDL-cholesterol and LDL-cholesterol both increased from baseline to 2 years, whereas no changes were observed in the UC group (Table 2). No changes in total cholesterol were observed in either the CCI or UC group. At 2 years, the CCI group had higher HDL-cholesterol, higher LDL-cholesterol, and lower triglycerides than UC. No between-group differences were observed at 2 years in systolic or diastolic blood pressure or total cholesterol (Table 2).

### Liver-Related Outcomes

Reductions were observed in liver-related outcomes including ALT (Figure 4E), AST, ALP, NLF and NFS in the CCI group, whereas no changes were observed in the UC group (Table 2, e.g., ALT; Supplementary Figure 3C). No Bonferroni-corrected group differences were observed for bilirubin, ALT, or AST at 2 years (Table 2).

### Kidney, Thyroid, and Inflammation Outcomes

The eGFR increased in the CCI but not UC group at 2 years (Table 2). The UC but not CCI group had increased anion gap and decreased uric acid. No bonferroni-corrected within-group changes in BUN, serum creatinine, TSH, or Free T4 were observed in either the CCI or UC group from baseline to 2 years. No between-group differences were observed for any thyroid- or kidney-related markers at 2 years (Table 2).

From baseline to 2 years, decreases in the CCI group's hsCRP (Figure 4F) and white blood cells were observed. No changes were observed in the UC group (Supplementary Figure 3D). At 2 years, both markers of inflammation were lower in the CCI group compared to the UC group (Table 2).

### Related Comorbidities

All within-group changes and between-group differences in comorbidities status among the CCI and UC group participants appear in Supplementary Table 4 (intent-to-treat analyses were conducted, all below ns = 262) and Supplementary Table 5 (per-protocol analyses). At 2 years, 27.2% of CCI participants (*P* = 4.9 × 10^−15^) and 6.5% of UC patients showed resolution of metabolic syndrome. The proportion of CCI patients with suspected steatosis was reduced from 95.8 to 67.4% (*P* < 0.0 × 10^−36^), whereas no change occurred in UC at 2 years. The proportion of patients without suspected fibrosis increased from 18.3 to 30.8% (*P* = 1.4 × 10^−5^) in the CCI, but did not change in the UC at 2 years.

### Adverse Events

In the CCI group, there were no reported adverse events between 1 and 2 years related to the intervention or that resulted in discontinuation, including no reported episodes of ketoacidosis or severe hypoglycemia requiring assistance. Limited or no change in kidney and thyroid functions were seen in the CCI at 2 years. Adverse events occurring in the first year of intervention (*n* = 6) were previously reported (11); during the second year of intervention, nine adverse events were reported including: one breast cancer diagnosis, one mycosis fungoides, one onset of atrial fibrillation (Afib) with heart failure, one onset of migraine, two cases of chest pain (one resulting in stent placement), one pulmonary effusion, and two pulmonary embolisms (one following orthopedic surgery and one with benign ovarian mass/Afib) in the CCI group. In the UC group, adverse events occurring in the first year (*n* = 6) were previously reported (11), and in the second year, adverse events occurred in six participants: one death from liver cancer, one hospitalization from recurrent seizure, one ureteropelvic junction obstruction from kidney stone, one cerebrovascular accident with left side weakness and sensory disturbances, one chest pain requiring percutaneous coronary intervention, and one deep vein thrombosis.

## Discussion

Following 2 years of a remote continuous care intervention supporting medical and lifestyle changes, the CCI participants demonstrated improved HbA1c, fasting glucose and insulin, and HOMA-IR. Pharmaceutical interventions of 1.5 to 3 years duration report HbA1c reductions of 0.2 to 1.0% with DPP-4 inhibitors, SGLT-2 inhibitors and GLP-1 agonists (6, 7, 49). The HbA1c reduction of 0.9% with this CCI is comparable to that observed in pharmaceutical trials, but is achieved while discontinuing 67.0% of diabetes-specific prescriptions including most insulins and all sulfonylureas that engender risks for weight gain and hypoglycemia (50, 51). Comparable improvements in glycemic control and reduced medication were not observed in UC participants recruited from the same healthcare system, suggesting that the CCI improves diabetes management relative to usual care. Other interventions using carbohydrate restriction reported variable long-term glycemic improvement outcomes (52–57). The 0.9% absolute (12% relative) HbA1c reduction observed at 2 years is consistent with low carbohydrate studies reporting HbA1c reductions of 8–15% at 2 to 3.5 years (52, 55–57) with medication reduction. Two other studies reported no changes in HbA1c from baseline to 2 years, even though the low-carbohydrate arm reduced HbA1c in the first 6 months (53, 54).

Criticisms of low-carbohydrate diets relate to poor adherence and long-term sustainability (25, 26, 28). In this CCI, self-monitoring combined with continuous remote-monitoring and feedback from the care team, including behavioral support and nutrition advice via the app, may have improved accountability and engagement (58). In addition to glucose and weight tracking, dietary adherence was monitored by blood ketones. The 2 year BHB increase above baseline demonstrates sustained dietary modification. While laboratory BHB levels were increased from baseline, the encouraged range of nutritional ketosis (≥0.5 mM) was observed in only a minority (14.1%) of participants at 2 years. On average, patient-measured BHB was ≥0.5 mM for 32.8% of measurements over the 2 years (Supplementary Figure 4). This reveals an opportunity to increase adherence to nutritional ketosis for patients not achieving their desired health outcomes while prompting future research investigating the association between dietary adherence and health improvements.

A majority of the CCI participants (53.5%) met criteria for diabetes reversal at 2 years while 17.6% achieved diabetes remission (i.e., glycemic control without medication use) based on intent-to-treat with multiple imputation. The percentage of all CCI enrollees (*N* = 262) with verified reversal and remission requiring both completion of 2 years of the trial and an obtained laboratory value for HbA1c were 37.8 and 14.9%, respectively. CCI diabetes reversal exceeds remission as prescriptions for metformin were usually continued given its role in preventing disease progression (10, 59), preserving β-cell function (59) and in the treatment of pre-diabetes per guidelines (28). Partial and complete remission rates of 2.4 and 0.2% per year, respectively, were reported in 122,781 T2D patients receiving standard diabetes care (4). The 2 year remission rate (both partial and complete) in the CCI (17.6%) is higher than that achieved through intensive lifestyle intervention (ILI) in the Look AHEAD trial (9.2%) (5). Greater diabetes remission in the CCI vs. Look AHEAD ILI could result from differences in the dietary intervention (23), patients' ability to self-select their lifestyle change or effectiveness of continuous remote care. Length of time with a T2D diagnosis is a factor in remission, with longer time since diagnosis resulting in lower remission (4, 5, 9). Despite a mean and median of 8.4 and 7 years since diagnosis among CCI participants, the remission rate was higher than the Look AHEAD trial where its participants had a median of 5 years (4) since diabetes diagnosis.

Participants in the CCI achieved 10% mean weight loss (−11.9 kg) at 2 years. CCI weight loss was comparable to observed weight loss following surgical gastric banding (−10.7 kg) at 2 years (59). Previous studies consistently report that weight loss increases the likelihood of T2D remission (4, 5, 9). CCI participants also improved blood pressure, triglycerides, and HDL-cholesterol. Total cholesterol was unchanged and LDL-cholesterol was increased at 2 years, but was not different from the LDL-cholesterol level observed at 1 year (+0.51 mg/dL, *P* = 0.85). Despite the rise in LDL-cholesterol, the CCI cohort improved in 22 out of 26 CVD markers at 1 year (33). These changes included a decrease in small LDL-particles and large VLDL-P and an increase in LDL-particle size partitioning with no changes in ApoB (33), a marker considered a better predictor of CVD risk than LDL-cholesterol (33, 60). Non-elevated LDL cholesterol values together with higher triglycerides and lower HDL-cholesterol are common in patients with abdominal obesity, T2D, and metabolic syndrome (61, 62); these individuals often still have elevated atherogenic lipoproteins such as non-HDL (63), small LDL particles (62, 64), and VLDL (62, 64). In the CCI group, non-HDL cholesterol did not change from baseline to 2 years (141.7 ± 2.6 at baseline to 143.7 ± 3.1 mg/dl, *P* = 0.51) and several cardiovascular risk factors improved, suggesting that the rise in LDL-cholesterol may not be associated with increased atherogenic risk (65).

The CCI group had a reduction in visceral fat content, CAF and A/G ratio. This is consistent with other low-carbohydrate interventions reporting visceral fat reduction as a component of weight loss (30, 56, 66–68). Anatomical distribution of fat around the abdominal area (“android” obesity) is associated with T2D (69) and other comorbidities such as metabolic syndrome (70) and NAFLD (71). The alleviation of visceral fat in the CCI group was concurrent with resolution of metabolic syndrome at 2 years, while sustaining 1 year improvements of liver enzymes (10), steatosis, and fibrosis (72). The comprehensive effect of reduced visceral fat and improvement in associated comorbidities was previously reported (68, 73, 74). Rat studies have shown that removal of visceral adipose tissue increases insulin sensitivity while delaying T2D (75), and prevents metabolic syndrome and NAFLD (76). Resolution of liver steatosis and fibrosis may protect against other T2D macrovascular and microvascular complications such as cardiovascular disease and nephropathy (77). Furthermore, abdominal adiposity and NAFLD are frequently associated with altered inflammatory pathways in T2D patients (71). Excess free fatty acids from visceral adipose tissue may initiate chronic low-grade inflammation and activate nuclear factor kappa B signaling (71, 77). CCI participants also improved inflammatory status (hsCRP and WBC) at 1 year (10) and 2 years.

While some studies in animal models (78, 79) and children treated with ketogenic diets (80, 81) have suggested retardation in skeletal development and reduction in BMD, in this study of adults with T2D the CCI group had no change in total spine BMD over 2 years. Our results are consistent with other adult ketogenic dietary studies that reported no bone mass loss in short-term (66) or long-term follow-up of 2 (67, 82) and 5 (83) years. The differing findings of ketogenic diet on bone mass between adults and children could be due to differential effects on developed and mineralized vs. developing bones (84). In this study, the CCI group had a reduction (7.0%, 1.3 kg) in the calculated LELM. Most lean mass loss was encountered in the first year without further reduction in year 2. Studies have reported that obese adults have about 20% higher thigh muscle mass than those with normal weight (85). The reduced upper body load burden achieved through weight loss might explain the reduction of LELM. This reflects an appropriate weight loss-related reduction in muscle mass rather than muscle deficiency (86, 87). Weight loss (~10%) induced by energy restriction resulted in slightly higher lean mass loss than the CCI (8.4% appendicular lean mass and 7.6% total lean mass loss at 20 weeks) (88). Total lean mass loss from 10% weight reduction by bariatric surgery is reported in the range of 7.3 to 15.9% from baseline (89, 90). Greater weight is associated with more lean mass loss (91, 92). Approximately 25% of diet-induced weight loss (without exercise) often arises from lean mass (93). In the present intervention, lean mass loss contributed an estimated 14% to the lower extremity weight loss. The lower proportion of lean mass loss in the CCI group, despite higher percentage of weight loss, may be due to the adequate dietary protein recommendations (94, 95). Since ~73% of lean mass is water, the observed reduction of LELM in the first year of intervention may have arisen from natriuresis and water loss that occurs during keto-adaptation (96, 97).

### Strengths and Limitations

This study's strengths include its size and prospective, longitudinal data collection from two participant groups (CCI and UC) which allowed statistical analysis by linear mixed effects model to investigate intervention time and treatment effects. The UC group was prospectively recruited from the same healthcare system. While not randomized, the participants' self-selection of intervention may contribute to the observed high retention and predicts real-life clinical management of chronic disease. The study also included patients prescribed insulin and with long-standing disease, groups often excluded from prior studies. The multi-component aspect of the intervention involving regular biomarker monitoring and access to a remote care team may have improved the long-term dietary adherence and engagement. The dietary advice including encouraging participants to restrict carbohydrates, moderate protein intake, and eat to satiety may also help in maintaining long-term effectiveness. Weaknesses of this study include the lack of randomization and racial diversity limiting generalization of the results to all T2D patients. Interpretation of DXA body composition was limited to subregion analyses due to the scanner not accommodating the patients' complete body.

## Conclusions

At 2 years, the CCI, including remote medical management with instruction in nutritional ketosis, was associated with improvements in blood glucose, insulin, HbA1c, weight, blood pressure, triglyceride, liver function, and inflammation and reduced dependence upon medication. These long-term benefits were achieved concurrent with reduced prevalence of metabolic syndrome, and visceral adiposity. The CCI had no adverse effect on bone mineral density. The CCI group also had a higher prevalence of diabetes reversal and remission compared to the UC group following a standard diabetes care program. These results provide evidence that sustained improvement in diabetes status can be achieved through the continuous remote monitoring and accountability mechanisms provided by this multi-component CCI including recommendations for low-carbohydrate nutrition.

## Ethics Statement

This study was carried out in accordance with the recommendations of Franciscan Health Lafayette Institutional Review Board with written informed consent from all subjects. All subjects gave written informed consent in accordance with the Declaration of Helsinki. The protocol was approved by the Franciscan Health Lafayette Institutional Review Board.

## Author Contributions

SA, RA, and JM drafted the manuscript. SA, RA, AM, NB, and SH participated in data acquisition and compiling. RA and SA analyzed the data. JM, AM, NB, WC, RA, SA, SH, SP, and JV edited the manuscript. All authors approved the final version of the manuscript.

### Conflict of Interest Statement

SA, RA, SH, AM, NB, SP, and JM are employed by Virta Health Corp and were offered stock options. SP and JV are founders of Virta Health Corp. The remaining author declares that the research was conducted in the absence of any commercial or financial relationships that could be construed as a potential conflict of interest.

## Abbreviations

CCI: continuous care intervention
UC: usual care
T2D: type 2 diabetes
HbA1c: hemoglobin A1c
CVD: cardiovascular disease
VLCD: very low calorie diet
BMI: body mass index
BHB: beta-hydroxybutryrate
BMD: bone mineral density
CAF: central abdominal fat
A/G: android:gynoid ratio
LELM: lower extremity lean mass
HDL: high density lipoprotein
LDL: low density lipoprotein
LDL-C: LDL cholesterol
LDL-P: LDL particle
ALT: alanine aminotransferase
AST: aspartate aminotransferase
ALP: alkaline phosphatase
NAFLD: nonalcoholic fatty liver disease
NLF: NAFLD liver fat score
NFS: NAFLD fibrosis score
TSH: thyroid stimulating hormone
BUN: blood urea nitrogen
eGFR: estimated glomerular filtration rate
hsCRP: high sensitive C-reactive protein
WBC: white blood cells
HOMA-IR: Homeostatic Model Assessment of Insulin Resistance
SGLT-2: sodium-glucose cotransporter-2 inhibitors
DPP-4: dipeptidyl peptidase-4 inhibitors
GLP-1: glucagon-like-peptide 1 receptor agonists
FFM: fat-free mass
VAT: visceral adipose tissue
GLM: generalized linear model
LMM: linear mixed-effect model
ADA: American Diabetes Association
CLIA: Clinical Laboratory Improvement Amendments
IRB: Institutional Review Board
DXA: dual-energy X-ray absorptiometry.
