# Supplementary material for: Long-Term Effects of a Novel Continuous Remote Care Intervention Including Nutritional Ketosis for the Management of Type 2 Diabetes: A 2-Year Non-randomized Clinical Trial
Source: Front Endocrinol (Lausanne). 2019 Jun 5;10:348. doi: 10.3389/fendo.2019.00348 (PMC6561315; doi:10.3389/fendo.2019.00348)
Supplement: Supplementary file 1 [file Data_Sheet_1.pdf]

**Supplementary Data**

**Table of contents**

Supplementary Table 1……………………………………………………………………………………………………… 2

Supplementary Table 2……………………………………………………………………………………………………… 3

Supplementary Table 3……………………………………………………………………………………………………… 6

Supplementary Table 4………………………………………………………………………………………………………14

Supplementary Table 5………………………………………………………………………………………………………15

Supplementary Figure 1……………………………………………………………………………………………………17

Supplementary Figure 2……………………………………………………………………………………………………18

Supplementary Figure 3……………………………………………………………………………………………………19

Supplementary Figure 4……………………………………………………………………………………………………20

Supplementary References………………………………………………………………………………………………..21

TREND Statement Checklist ……………………………………………………………………………………………22

**Supplementary Table 1**

Equations for calculating HOMA-IR , LDL-cholesterol, NAFLD liver fat score and NAFLD fibrosis score

| Score | Equation |
|---|---|
| **HOMA-IR (insulin derived)** | [fasting insulin (mU/L) x fasting glucose (mg/dL)]/ 405 |
| **HOMA-IR (c-peptide derived)** | Calculation performed using spreadsheet downloaded from http://www.dtu.ox.ac.uk/homacalculator/ |
| **Friedewald LDL-cholesterol** | total cholesterol (mg/dL) - HDL cholesterol (mg/dL) - [TG (mg/dL)/5] |
| **NAFLD liver fat score (N-LFS)** | -2.89 + 1.18 x metabolic syndrome (yes=1 or no=0) + 0.45 x type 2 diabetes (yes=2 or no=0)* + 0.15 x fasting insulin (mU/l) + 0.04 x fasting serum AST (U/L) – 0.94 x AST/ALT |
| **NAFLD fibrosis score (NFS)** | −1.675 + 0.037 × Age (yrs) + 0.094 × BMI (kg/$m^2$) + 1.13 × IFG/diabetes (yes = 1, no = 0) + 0.99 × AST/ALT ratio − 0.013 × Platelet (×$10^9$/L) −0.66 × Albumin (g/dl) |

**Supplementary Table 2**

Criteria and cut-offs for diabetes reversal, diabetes partial and complete remission, metabolic syndrome, steatosis and absence of fibrosis

| Disease outcomes | Criteria and cut-offs used for assignment |
|---|---|
| **Diabetes reversal** | Sub-diabetic hyperglycemia and normoglycemia (HbA1c below 6.5%), without medications except metformin |
| **Diabetes partial remission (S1)** | Sub-diabetic hyperglycemia of at least 1 year duration, HbA1c level between 5.7-6.5%, without any medications (two HbA1c measurements) |
| **Diabetes complete remission (S1)** | Normoglycemia of at least 1 year duration, HbA1c below 5.7%, without any medications (two HbA1c measurements) |
| **Metabolic syndrome (S3,S4)** | Assigned according to the new International Diabetes Federation (IDF) and National Cholesterol Education Program's Adult Treatment Panel III (NCEP ATP III)] classification. Metabolic syndrome is assigned if any three of the following five factors were listed: |

| | |
|---|---|
| | 1) **Central obesity** defined using BMI and waist circumference: ≥ 40 inches for male and ≥ 37 inches for female. Those missing waist circumference information, if BMI ≥ 30kg/m$^2$, central obesity is assumed.<br>2) **Raised triglycerides**: ≥ 150 mg/dL (1.7 mmol/L)<br>3) **Reduced HDL-cholesterol**: < 40 mg/dL (1.03mmol/L) in males or < 50 mg/dL (1.29mmol/L) in females<br>4) **Raised fasting blood glucose**: ≥ 100 mg/dL<br>5) **Raised blood pressure**: systolic BP ≥ 130 or diastolic BP ≥ 85mmHg<br>For those with missing data:<br>A) If patient is missing more than two criteria from the five factors, he/she is classified as missing or no assignment.<br>B) If patient is missing two or fewer criteria excluding central obesity and any of the remaining criteria were classified positive (present); he/she is assigned as “having metabolic syndrome”<br>C) If patient is missing only one criteria excluding central obesity and if the remaining criteria were classified negative (not present), he/she is assigned as “not having metabolic syndrome”. |
| **Suspected steatosis (S4)** | Optimal cut-off point of > -0.640 predicts increased liver fat content (suspected steatosis) with sensitivity of 86% and specificity of 71%. |

| | |
|---|---|
| **Absence of fibrosis (S5)** | Optimal cut-off point of < -1.455 predicts absence of significant fibrosis with a negative predictive value of 93%. |

**Supplementary Table 3**
Descriptives and results of completer-only analyses

| | Baseline | | | 1 Year | | | 2 Years | | | |
|---|---|---|---|---|---|---|---|---|---|---|
| | N | Mean (SD) or ±SE | Range | N | Mean (SD) or ±SE | Range | N | Mean (SD) or ±SE | Range | P |
| **Glycemic** | | | | | | | | | | |
| Hemoglobin A1c (%)[a] | | | | | | | | | | |
| CCI-all education | 262 | 7.6(1.5) | 5.3-13.6 | 204 | 6.2(0.9) | 4.50-12.0 | 183 | 6.6(1.3) | 4.8-12.5 | $9.9 \times 10^{-16}$ |
| Usual Care | 87 | 7.6(1.8) | 5.1-12.5 | 76 | 7.9(1.8) | 5.3-13.6 | 68 | 8.2(2.0) | 5.6-13.8 | 0.01 |
| CCI-all vs. usual care | | | | | | | | | | $1.9 \times 10^{-10}$ |
| C-Peptide (nmol $L^{-1}$)[a] | | | | | | | | | | |
| CCI-all education | 248 | 4.4(2.2) | 0.01-12.4 | 196 | 3.4(1.8) | 0.01-12.4 | 173 | 3.3(1.7) | 0.01-11.4 | $1.5 \times 10^{-15}$ |
| Usual Care | 79 | 4.2(2.5) | 0.3-11.2 | 63 | 4.3(2.8) | 0.3-15.3 | 57 | 3.4(1.9) | 0.3-7.4 | 0.76 |
| CCI-all vs. usual care | | | | | | | | | | 0.25 |
| Fasting glucose (mg/dL)[a] | | | | | | | | | | |
| CCI-all education | 258 | 160.8(61.4) | 70.0-418.0 | 205 | 124.0(35.2) | 71.0-318.0 | 179 | 131.1(44.8) | 42.0-363.0 | $5.9 \times 10^{-8}$ |
| Usual Care | 86 | 156.2(72.6) | 40.0-356.0 | 76 | 166.9(83.0) | 50.0-514.0 | 67 | 181.2(90.1) | 65.0-466.0 | 0.10 |
| CCI-all vs. usual care | | | | | | | | | | $3.6 \times 10^{-6}$ |
| Fasting Insulin (mIU $L^{-1}$)[a,c] | | | | | | | | | | |
| CCI-all education | 248 | 28.6(23.9) | 2.5-209.5 | 196 | 18.0(24.2) | 0.9-285.7 | 172 | 17.5(25.2) | 0.6-312.4 | $1.9 \times 10^{-15}$ |
| Usual Care | 79 | 29.1(24.9) | 0.4-122.6 | 63 | 30.8(33.7) | 2.3-205.1 | 57 | 23.0(18.7) | 4.3-114.5 | 0.98 |
| CCI-all vs. usual care | | | | | | | | | | 0.004 |
| HOMA-IR (insulin derived), all[a,c] | | | | | | | | | | |
| CCI-all education | 220 | 9.0(6.2) | 1.0-42.4 | 181 | 4.8(3.7) | 0.7-20.4 | 162 | 5.9(9.9) | 0.1-118.0 | $2.1 \times 10^{-12}$ |
| Usual Care | 78 | 10.6(9.1) | 0.05-44.7 | 61 | 12.7(12.6) | 0.4-52.6 | 56 | 10.4(9.3) | 1.2-39.3 | 0.28 |
| CCI-all vs. usual care | | | | | | | | | | |

| | | | | | | | | | | |
|---|---|---|---|---|---|---|---|---|---|---|
| | | | | | | | | | | 2.2 x $10^{-5}$ |
| HOMA-IR (insulin derived), excluding exogenous users[a,c]<br>CCI-all education<br>Usual Care<br>CCI-all vs. usual care | 157<br>42 | 8.8(5.6)<br>9.4(8.3) | 1.0-35.2<br>1.3-41.5 | 156<br>28 | 4.6(3.5)<br>13.2(14.2) | 0.7-18.8<br>1.5-51.7 | 143<br>22 | 6.0(10.3)<br>8.4(7.6) | 0.2-118.0<br>1.2-34.0 | 0.003<br>0.24<br>0.01 |
| HOMA-IR (C-peptide derived), all[a,c]<br>CCI-all education<br>Usual Care<br>CCI-all vs. usual care | 244<br>78 | 11.7(7.4)<br>11.1(7.6) | 0.04-66.7<br>0.6-45.5 | 190<br>60 | 8.1(4.4)<br>12.5(10.7) | 0.05-32.3<br>0.6-66.7 | 164<br>55 | 8.0(4.2)<br>12.6(19.5) | 0.03-27.8<br>0.5-142.9 | 5.4 x $10^{-14}$<br>0.60<br>0.02 |
| **Metabolic and Body Composition** | | | | | | | | | | |
| Weight-clinic (kgs)[a]<br>CCI-all education<br>Usual Care<br>CCI-all vs. usual care | 257<br>83 | 116.5(25.9)<br>105.6(22.1) | 63.4-215.6<br>71.0-170.6 | 187<br>73 | 101.1(22.2)<br>109.3(24.5) | 55.4-166.7<br>74.6-172.8 | 147<br>53 | 102.5(21.9)<br>110.5(25.2) | 58.5-181.0<br>71.2-166.5 | 4.6 x $10^{-26}$<br>0.35<br>2.7 x $10^{-5}$ |
| Spine bone mineral density ($g/cm^2$)[a]<br>CCI-all education | 238 | 1.2(0.2) | 0.8-1.8 | 195 | 1.2(0.2) | 0.9-1.7 | 167 | 1.2(0.2) | 0.8-1.8 | 0.01 |
| Central abdominal fat (kg)[a]<br>CCI-all education | 237 | 5.8(1.7) | 1.9-10.8 | 195 | 4.6(1.7) | 1.3-9.7 | 167 | 4.9(1.7) | 1.5-10.1 | 1.9 x $10^{-22}$ |
| Android: gynoid ratio[a]<br>CCI-all education | 238 | 1.3(0.3) | 0.7-2.5 | 195 | 1.2(0.3) | 0.7-2.3 | 167 | 1.2(0.3) | 0.7-2.4 | 1.6 x $10^{-6}$ |
| Lean leg mass (kg)[a]<br>CCI-all education | 238 | 18.5(4.1) | 10.3-30.1 | 195 | 17.6(4.4) | 10.6-33.7 | 167 | 17.3(4.2) | 10.4-34.6 | 1.2 x $10^{-23}$ |
| **Cardiovascular** | | | | | | | | | | |

| | | | | | | | | | | |
|---|---|---|---|---|---|---|---|---|---|---|
| Systolic blood pressure (mmHg)[a] | | | | | | | | | | |
| CCI-all education | 260 | 131.9(14.1) | 92.0-180.0 | 188 | 125.7(11.9) | 92.0-160.0 | 150 | 126.1(13.1) | 92.0-160.0 | $1.8 \times 10^{-5}$ |
| Usual Care | 79 | 129.8(13.6) | 102.0-170.0 | 73 | 129.1(15.3) | 102.0-170.0 | 53 | 129.9(11.1) | 102.0-152.0 | 0.92 |
| CCI-all vs. usual care | | | | | | | | | | 0.03 |
| Diastolic blood pressure (mmHg)[a] | | | | | | | | | | |
| CCI-all education | 260 | 82.1(8.3) | 60.0-110.0 | 188 | 78.0(7.5) | 56.0-100.0 | 150 | 78.7(8.0) | 60.0-100.0 | $1.5 \times 10^{-4}$ |
| Usual Care | 79 | 82.0(8.9) | 62.0-110.0 | 72 | 81.3(9.5) | 48.0-100.0 | 53 | 81.7(7.2) | 62.0-96.0 | 0.95 |
| CCI-all vs. usual care | | | | | | | | | | 0.01 |
| Total cholesterol (mg/dL)[a] | | | | | | | | | | |
| CCI-all education | 247 | 183.6(41.2) | 97.0-349.0 | 196 | 190.2(45.1) | 105.0-320.0 | 171 | 193.4(43.6) | 106.0-320.0 | 0.004 |
| Usual Care | 79 | 183.8(45.8) | 91.0-339.0 | 63 | 180.2(61.1) | 94.0-404.0 | 56 | 181.8(57.0) | 102.0-430.0 | 0.82 |
| CCI-all vs. usual care | | | | | | | | | | 0.13 |
| LDL-cholesterol (mg/dL)[a] | | | | | | | | | | |
| CCI-all education | 232 | 102.5(32.9) | 29.0-211.0 | 188 | 112.3(38.3) | 30.0-240.0 | 162 | 114.7(38.4) | 36.0-231.0 | $9.4 \times 10^{-5}$ |
| Usual Care | 70 | 101.5(36.2) | 29.0-204.0 | 53 | 89.3(29.5) | 29.0-159.0 | 50 | 93.9(32.3) | 36.0-165.0 | 0.12 |
| CCI-all vs. usual care | | | | | | | | | | $7.4 \times 10^{-4}$ |
| HDL-cholesterol (mg/dL)[a] | | | | | | | | | | |
| CCI-all education | 247 | 42.2(13.4) | 12.0-117.0 | 196 | 50.1(15.9) | 15.0-111.0 | 170 | 51.1(15.8) | 23.0-96.0 | $2.8 \times 10^{-15}$ |
| Usual Care | 79 | 37.6(11.2) | 15.0-66.0 | 63 | 35.9(12.3) | 13.0-77.0 | 56 | 42.3(10.3) | 21.0-65.0 | 0.11 |
| CCI-all vs. usual care | | | | | | | | | | 0.02 |
| Triglycerides (mg/dL)[a,d] | | | | | | | | | | |
| CCI-all education | 247 | 197.2(143.4) | 46.0-1432.0 | 196 | 148.9(141.8) | 41.0-1308.0 | 170 | 153.3(135.5) | 42.0-1356.0 | $9.2 \times 10^{-9}$ |
| Usual Care | 79 | 282.9(401.2) | 84.0-2781.0 | 63 | 314.5(487.7) | 78.0-3639.0 | 56 | 209.5(138.7) | 74.0-708.0 | 0.80 |
| CCI-all vs. usual care | | | | | | | | | | 0.01 |

| | | | | | | | | | | |
|---|---|---|---|---|---|---|---|---|---|---|
| **Liver** | | | | | | | | | | |
| ALT (Units/L[1])[a,c] | | | | | | | | | | |
| CCI-all education | 257 | 30.7(22.8) | 7.0-258.0 | 205 | 21.8(11.7) | 7.0-111.0 | 179 | 22.5(11.5) | 7.0-99.0 | $2.0 \times 10^{-9}$ |
| Usual Care | 86 | 27.7(19.8) | 8.0-153.0 | 75 | 28.3(20.3) | 7.0-103.0 | 66 | 28.9(19.1) | 7.0-112.0 | 0.44 |
| CCI-all vs. usual care | | | | | | | | | | 0.05 |
| AST (Units/L)[a,c] | | | | | | | | | | |
| CCI-all education | 257 | 23.7(15.2) | 7.0-130.0 | 205 | 19.1(6.9) | 8.0-73.0 | 178 | 19.5(6.3) | 10.0-59.0 | $2.0 \times 10^{-4}$ |
| Usual Care | 86 | 23.9(19.4) | 9.0-156.0 | 74 | 24.6(16.2) | 10.0-120.0 | 66 | 24.9(14.8) | 12.0-79.0 | 0.15 |
| CCI-all vs. usual care | | | | | | | | | | 0.005 |
| ALP (Units/L)[a] | | | | | | | | | | |
| CCI-all education | 256 | 74.1(22.1) | 25.0-172.0 | 205 | 64.8(21.2) | 27.0-174.0 | 178 | 64.0(19.6) | 28.0-160.0 | $1.2 \times 10^{-14}$ |
| Usual Care | 86 | 77.4(26.3) | 25.0-154.0 | 74 | 78.7(26.7) | 35.0-169.0 | 66 | 81.5(31.1) | 32.0-179.0 | 0.08 |
| CCI-all vs. usual care | | | | | | | | | | $3.5 \times 10^{-7}$ |
| Bilirubin (mg/dL)[a,c] | | | | | | | | | | |
| CCI-all education | 256 | 0.5(0.2) | 0.2-1.6 | 205 | 0.5(0.2) | 0.2-2.1 | 178 | 0.5(0.3) | 0.2-2.3 | 0.39 |
| Usual Care | 86 | 0.6(0.3) | 0.2-1.5 | 74 | 0.6(0.3) | 0.2-1.7 | 66 | 0.6(0.4) | 0.2-2.5 | 0.14 |
| CCI-all vs. usual care | | | | | | | | | | 0.72 |
| NAFLD-Liver fat score[a,c] | | | | | | | | | | |
| CCI-all education | 243 | 3.4(3.8) | -2.6-30.9 | 184 | 1.5(3.9) | -1.9-42.8 | 142 | 0.9(4.3) | -3.4-45.3 | $1.1 \times 10^{-20}$ |
| Usual Care | 74 | 3.1(3.6) | -2.0-16.0 | 59 | 4.6(5.4) | -1.0-30.7 | 44 | 2.7(3.3) | -1.2-16.4 | 0.10 |
| CCI-all vs. usual care | | | | | | | | | | $1.5 \times 10^{-4}$ |
| NAFLD-Fibrosis score[a] | | | | | | | | | | |
| CCI-all education | 238 | -0.2(1.4) | -4.0-5.1 | 173 | -0.8(1.1) | -3.3-2.7 | 132 | -0.7(1.2) | -3.8-4.7 | $1.1 \times 10^{-10}$ |
| Usual Care | 75 | -0.8(1.4) | -4.6-2.1 | 60 | -0.4(1.5) | -4.6-2.3 | 40 | -0.2(1.4) | -4.7-2.4 | 0.13 |
| CCI-all vs. usual care | | | | | | | | | | $1.7 \times 10^{-4}$ |
| **Kidney** | | | | | | | | | | |

| | | | | | | | | | | |
|---|---|---|---|---|---|---|---|---|---|---|
| Anion gap (mmol $L^{-1}$)[a]<br>CCI-all education<br>Usual Care<br>CCI-all vs. usual care | 257<br>86 | 6.8(1.7)<br>6.9(1.8) | 2.0-12.0<br>3.0-12.0 | 205<br>76 | 7.1(1.8)<br>7.8(1.9) | 2.0-12.0<br>4.0-13.0 | 179<br>66 | 7.2(1.6)<br>7.7(1.9) | 3.0-12.0<br>4.0-13.0 | $4.9 \times 10^{-4}$<br>$1.8 \times 10^{-4}$<br>0.08 |
| BUN (mg/dL)[a,c]<br>CCI-all education<br>Usual Care<br>CCI-all vs. usual care | 258<br>86 | 16.9(6.6)<br>16.1(6.2) | 7.0-70.0<br>5.0-36.0 | 205<br>76 | 19.0(7.8)<br>16.0(5.8) | 8.0-86.0<br>6.0-44.0 | 179<br>67 | 17.8(6.6)<br>16.4(6.8) | 7.0-57.0<br>6.0-49.0 | 0.05<br>0.86<br>0.15 |
| eGFR (mL $s^{-1}$ $m^{-2}$)[a]<br>CCI-all education<br>Usual Care<br>CCI-all vs. usual care | 258<br>86 | 80.5(13.6)<br>79.2(13.7) | 26.0-90.0<br>33.0-90.0 | 205<br>76 | 82.7(12.0)<br>80.1(13.0) | 31.0-90.0<br>29.0-90.0 | 178<br>66 | 83.0(11.4)<br>79.1(14.9) | 40.0-90.0<br>21.0-90.0 | $9.9 \times 10^{-4}$<br>0.84<br>0.02 |
| Serum creatinine (mg/dL)[a,c]<br>CCI-all education<br>Usual Care<br>CCI-all vs. usual care | 258<br>86 | 0.9(0.2)<br>0.9(0.2) | 0.5-2.2<br>0.5-2.2 | 205<br>76 | 0.8(0.2)<br>0.9(0.2) | 0.4-1.9<br>0.5-1.9 | 179<br>66 | 0.8(0.2)<br>0.9(0.4) | 0.5-1.8<br>0.6-3.2 | 0.004<br>0.76<br>0.15 |
| Uric acid (mg/dL)[a]<br>CCI-all education<br>Usual Care<br>CCI-all vs. usual care | 261<br>85 | 5.9(1.5)<br>5.6(1.5) | 2.7-10.2<br>2.9-10.5 | 203<br>72 | 5.9(1.5)<br>5.4(1.4) | 1.7-10.5<br>2.9-9.0 | 179<br>55 | 5.8(1.5)<br>5.0(1.2) | 2.9-10.1<br>2.6-8.0 | 0.19<br>0.003<br>0.002 |
| **Thyroid** | | | | | | | | | | |
| TSH (mIU $L^{-1}$)[a,c]<br>CCI-all education<br>Usual Care<br>CCI-all vs. usual care | 259<br>86 | 2.3(1.7)<br>3.8(17.1) | 0.03-15.3<br>0.03-159.9 | 203<br>74 | 1.9(1.1)<br>4.8(23.9) | 0.02-8.1<br>0.1-207.8 | 179<br>60 | 2.0(1.2)<br>2.9(6.2) | 0.2-10.9<br>0.03-49.3 | 0.08<br>0.79<br>0.31 |

| | | | | | | | | | | |
|---|---|---|---|---|---|---|---|---|---|---|
| Free T4 (ng/dL)[a,c] | | | | | | | | | | |
| CCI-all education | 260 | 0.9(0.2) | 0.6-1.9 | 203 | 0.9(0.2) | 0.6-1.8 | 179 | 0.9(0.2) | 0.6-1.8 | 0.34 |
| Usual Care | 86 | 0.9(0.3) | 0.4-3.0 | 73 | 0.9(0.2) | 0.2-1.8 | 57 | 0.9(0.3) | 0.6-2.8 | 0.03 |
| CCI-all vs. usual care | | | | | | | | | | 0.47 |
| **Other** | | | | | | | | | | |
| Beta-hydroxybutyrate (mmol $L^{-1}$)[a,c] | | | | | | | | | | |
| CCI-all education | 248 | 0.2(0.2) | 0.04-1.1 | 196 | 0.3(0.3) | 0.04-2.3 | 170 | 0.3(0.4) | 0.05-2.7 | $1.1 \times 10^{-5}$ |
| Usual Care | 79 | 0.2(0.1) | 0.05-0.7 | 63 | 0.2(0.2) | 0.04-1.5 | 55 | 0.2(0.3) | 0.04-1.4 | 0.17 |
| CCI-all vs. usual care | | | | | | | | | | 0.09 |
| hsC-reactive protein (nmol $L^{-1}$)[a,c] | | | | | | | | | | |
| CCI-all education | 249 | 8.5(14.5) | 0.5-207.5 | 203 | 5.6(6.9) | 0.2-42.4 | 179 | 6.1(9.7) | 0.2-87.4 | $1.6 \times 10^{-12}$ |
| Usual Care | 85 | 8.9(8.6) | 0.4-35.6 | 71 | 10.4(14.6) | 0.3-103.5 | 55 | 8.3(8.5) | 0.4-30.7 | 0.30 |
| CCI-all vs. usual care | | | | | | | | | | 0.001 |
| White blood cell | | | | | | | | | | |
| (k/cumm)[a] | 260 | 7.2(1.9) | 3.5-13.3 | 205 | 6.5(1.8) | 2.7-13.0 | 180 | 6.6(2.0) | 2.4-14.5 | $9.0 \times 10^{-5}$ |
| CCI-all education | 86 | 8.1(2.4) | 3.6-14.7 | 75 | 8.2(2.4) | 2.9-13.8 | 60 | 8.0(2.6) | 4.1-19.3 | 0.85 |
| Usual Care | | | | | | | | | | $8.0 \times 10^{-5}$ |
| CCI-all vs. usual care | | | | | | | | | | |
| **Diabetes Medication** | | | | | | | | | | |
| Any diabetes medication, excluding | | | | | | | | | | |
| metformin (%)[b] | 262 | 56.9±3.1 | — | 218 | 28.0±3.1 | — | 194 | 26.8±3.2 | — | $1.3 \times 10^{-11}$ |
| CCI-all education | 87 | 66.7±5.1 | | 78 | 75.6±4.9 | | 58 | 79.3±5.4 | | 0.004 |
| Usual Care | | | | | | | | | | |
| Sulfonylurea (%)[b] | | | | | | | | | | |
| CCI-all education | 262 | 23.7±2.6 | — | 218 | 00.0±0.0 | — | 194 | 00.0±0.0 | — | $4.2 \times 10^{-12}$ |

| | | | | | | | | | | |
|---|---|---|---|---|---|---|---|---|---|---|
| Usual Care | 87 | 24.1±4.6 | | 78 | 25.6±5.0 | | 58 | 29.3±6.0 | | 0.23 |
| Insulin (%)[b] | | | | | | | | | | |
| CCI-all education | 262 | 29.8±2.8 | — | 218 | 14.7±2.4 | — | 194 | 11.3±2.3 | — | $9.1 \times 10^{-9}$ |
| Usual Care | 87 | 46.0±5.4 | | 78 | 51.3±5.7 | | 58 | 55.2±6.6 | | 0.23 |
| Thiazolidinedione (%)[b] | | | | | | | | | | |
| CCI-all education | 262 | 1.5±0.8 | — | 218 | 0.5±0.5 | — | 194 | 2.6±1.1 | — | 0.73 |
| Usual Care | 87 | 1.2±1.2 | | 78 | 1.3±1.3 | | 58 | 6.9±3.4 | | 0.25 |
| SGLT-2 (%)[b] | | | | | | | | | | |
| CCI-all education | 262 | 10.3±1.9 | — | 218 | 0.9±0.7 | — | 194 | 3.1±1.3 | — | 0.01 |
| Usual Care | 87 | 14.9±3.8 | | 78 | 16.7±4.3 | | 58 | 13.8±4.6 | | 0.69 |
| DPP-4 (%)[b] | | | | | | | | | | |
| CCI-all education | 262 | 9.9±1.9 | — | 218 | 6.4±1.7 | — | 194 | 6.7±1.8 | — | 0.42 |
| Usual Care | 87 | 8.1±2.9 | | 78 | 11.5±3.6 | | 58 | 8.6±3.7 | | 0.99 |
| GLP-1 (%)[b] | | | | | | | | | | |
| CCI-all education | 262 | 13.4±2.1 | — | 218 | 15.1±2.4 | — | 194 | 10.8±2.2 | — | 0.42 |
| Usual Care | 87 | 16.1±4.0 | | 78 | 20.5±4.6 | | 58 | 27.6±5.9 | | 0.18 |
| Metformin (%)[b] | | | | | | | | | | |
| CCI-all education | 262 | 71.4±2.8 | — | 218 | 64.2±3.3 | — | 194 | 63.9±3.5 | — | 0.05 |
| Usual Care | 87 | 60.9±5.3 | | 78 | 60.3±5.6 | | 58 | 63.8±6.4 | | 0.18 |

*Note.* All means and standard deviations or standard errors are without any adjustments and include all available data for the time point. Abbreviations: SD, standard deviation; CCI, continuous care intervention; UC, usual care; HOMA-IR, homeostatic model assessment of insulin resistance; LDL, low-density lipoprotein; HDL, high-density lipoprotein; ALT, alanine aminotransferase; AST, aspartate aminotransferase; ALP, alkaline phosphatase; NAFLD, nonalcoholic fatty liver disease; BUN, blood urea nitrogen; eGFR, estimated glomerular filtration rates; TSH, thyroid stimulating hormone; SGLT-2, Sodium glucose co-transporter 2 inhibitor; DPP-4, Dipeptidyl peptidase-4 inhibitor; GLP-1, Glucagon-like peptide 1 receptor agonist.

[a]P-values representing changes from baseline to 2 years and between group-differences at 2 years were obtained from linear mixed-effects models. Covariates in the model included baseline age, sex, race, body mass index, and insulin use. Only participants with both baseline and 2 year data for the outcome were included in the analysis.
[b]P-values representing changes in the proportions of participants taking medication from baseline to 2 years were obtained from McNemar’s tests, with continuity correction when appropriate. Only participants with both baseline and 2 year data for the medication were included in the analysis.
[c]Variable was positively skewed and after removing the top 1% of values, skew and kurtosis values fell within acceptable ranges. Analyses were conducted on data excluding the top 1% of values for each variable.
[d]Variable was positively skewed and a natural log transformation was performed. The linear mixed-effects model analysis including covariates was conducted on the transformed variable.

**Supplementary Table 4.**

Disease outcomes in CCI and UC participants after 2 years (Intent-to-treat analysis with imputation)

| Disease Outcomes | Continuous Care Intervention (n=262) | | | Usual Care (n=87) | | | Between group |
|---|---|---|---|---|---|---|---|
| | Baseline | 2 Years | P | Baseline | 2 Years | P | P |
| **Diabetes Reversal (%)** | 12.1±2.0 | 53.5±3.4 | $<0.0\times10^{-36}$ | 16.4±4.5 | 9.3±3.9 | 0.04 | $<0.0\times10^{-36}$ |
| **Diabetes Remission (%)[a]** | — | 17.6±2.5 | — | — | 2.4±1.7 | — | $5.1\times10^{-9}$ |
| **Complete Remission (%)** | — | 6.7±1.6 | — | — | 0.0±0.0 | — | $1.1\times10^{-5}$ |
| **Metabolic Syndrome (%)** | 89.1±2.0 | 61.9±4.0 | $4.9\times10^{-15}$ | 92.4±3.3 | 85.9±5.1 | 0.24 | $4.7\times10^{-7}$ |
| **Suspected Steatosis (%)** | 95.8±1.4 | 67.4±4.2 | $<0.0\times10^{-36}$ | 94.7±3.0 | 89.0±5.1 | 0.16 | $2.5\times10^{-7}$ |
| **Absence of Fibrosis (%)** | 18.3±2.5 | 30.8±4.0 | $1.4\times10^{-5}$ | 24.9±5.4 | 15.9±5.8 | 0.08 | $4\times10^{-3}$ |

*Note.* Percentages and standard errors are provided. Estimates were obtained from generalized estimating equation models which provide adjusted proportions, controlling for baseline age, sex, race, time since diagnosis, body mass index, and insulin use. Multiple imputation was used to replace missing values, facilitating intent-to-treat analyses. A significance level of $P<0.0012$ ensures overall simultaneous significance of $P < 0.05$ over the 43 study variables using Bonferroni correction.
[a]Diabetes remission includes both partial and complete remission.

**Supplementary Table 5.**
Disease outcomes in CCI and UC participants after 2 years (Completers-only analysis)

| Disease Outcomes | Continuous Care Intervention | | | | | Usual Care (n=87) | | | | | Between group |
|---|---|---|---|---|---|---|---|---|---|---|---|
| | N | Baseline | N | 2 Years | P | N | Baseline | N | 2 Years | P | P |
| **Diabetes Reversal (%)** | 262 | 12.2±2.0 | 181 | 54.7±3.7 | $<0.0 \times 10^{-36}$ | 87 | 20.7±4.4 | 57 | 10.5±4.1 | 0.07 | $5.4 \times 10^{-15}$ |
| **Diabetes Remission (%)**[a] | — | — | 208 | 18.8±2.7 | — | — | — | 79 | 2.5±1.8 | — | $1.6 \times 10^{-8}$ |
| **Complete Remission (%)** | — | — | 210 | 6.7±1.7 | — | — | — | 81 | 0.0±0.0 | — | $1.1 \times 10^{-4}$ |
| **Metabolic Syndrome (%)** | 262 | 88.6±2.0 | 154 | 63.0±3.9 | $9.9 \times 10^{-11}$ | 81 | 91.4±3.1 | 54 | 87.0±4.6 | 0.51 | $8.9 \times 10^{-5}$ |
| **Suspected Steatosis (%)** | 243 | 96.3±1.2 | 142 | 67.6±3.9 | $7.7 \times 10^{-13}$ | 74 | 94.6±2.6 | 44 | 88.6±4.8 | 0.40 | $3.9 \times 10^{-5}$ |
| **Absence of Fibrosis (%)** | 238 | 18.1±2.5 | 132 | 29.6±4.0 | 0.003 | 75 | 28.0±5.2 | 40 | 17.5±6.1 | 0.58 | 0.09 |

*Note.* All percentages and standard errors are without any adjustments and include all available data for the time point. P-values representing within-group changes from baseline to 2 years and between-group differences at 2 years were obtained from generalized estimating equation models. Covariates in the model included baseline age, sex, race, time since diagnosis, body mass index, and insulin use. Only participants with both baseline and 2 year data for the outcome were included in the analysis. A significance level of $P<0.0012$ ensures overall simultaneous significance of $P < 0.05$ over the 43 study variables using Bonferroni correction.
[a]Diabetes remission includes both partial and complete remission.

**Supplementary Figures Legend**

**Supplementary Figure 1.** Adjusted mean changes (CCI versus UC) from baseline to 2-years in (A) HbA1c, (B) Fasting insulin, (C) Weight.

**Supplementary Figure 2.** Stratification of participants based on weight change (%) categories in each intervention groups, UC and CCI, among completers. Category <5% includes participants with weight gain.

**Supplementary Figure 3.** Adjusted mean changes (CCI versus UC) from baseline to 2-years in (A) Systolic Blood Pressure, (B) Diastolic Blood Pressure, (C) Alanine aminotransferase (ALT), and (D) High sensitive C-reactive protein (hsCRP).

**Supplementary Figure 4**. Cumulative relative frequency (%) of percentage participants reporting BHB ≥ 0.5mM at first, second and both years of the study. The differences in the distribution of participants reporting BHB ≥ 0.5mM between one and two years are illustrated in the figure.

## Supplementary Figure 1

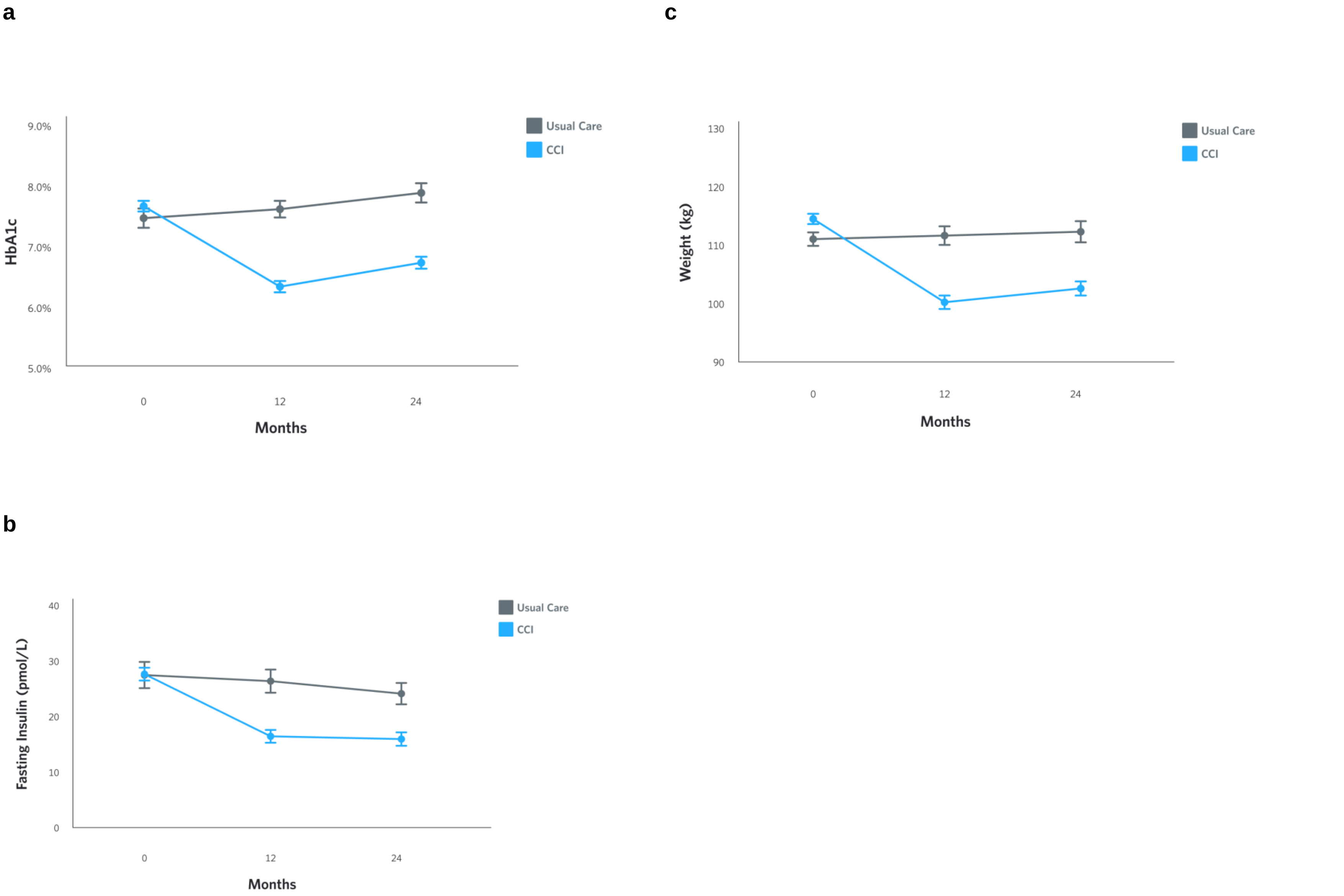

**Supplementary Figure 2**

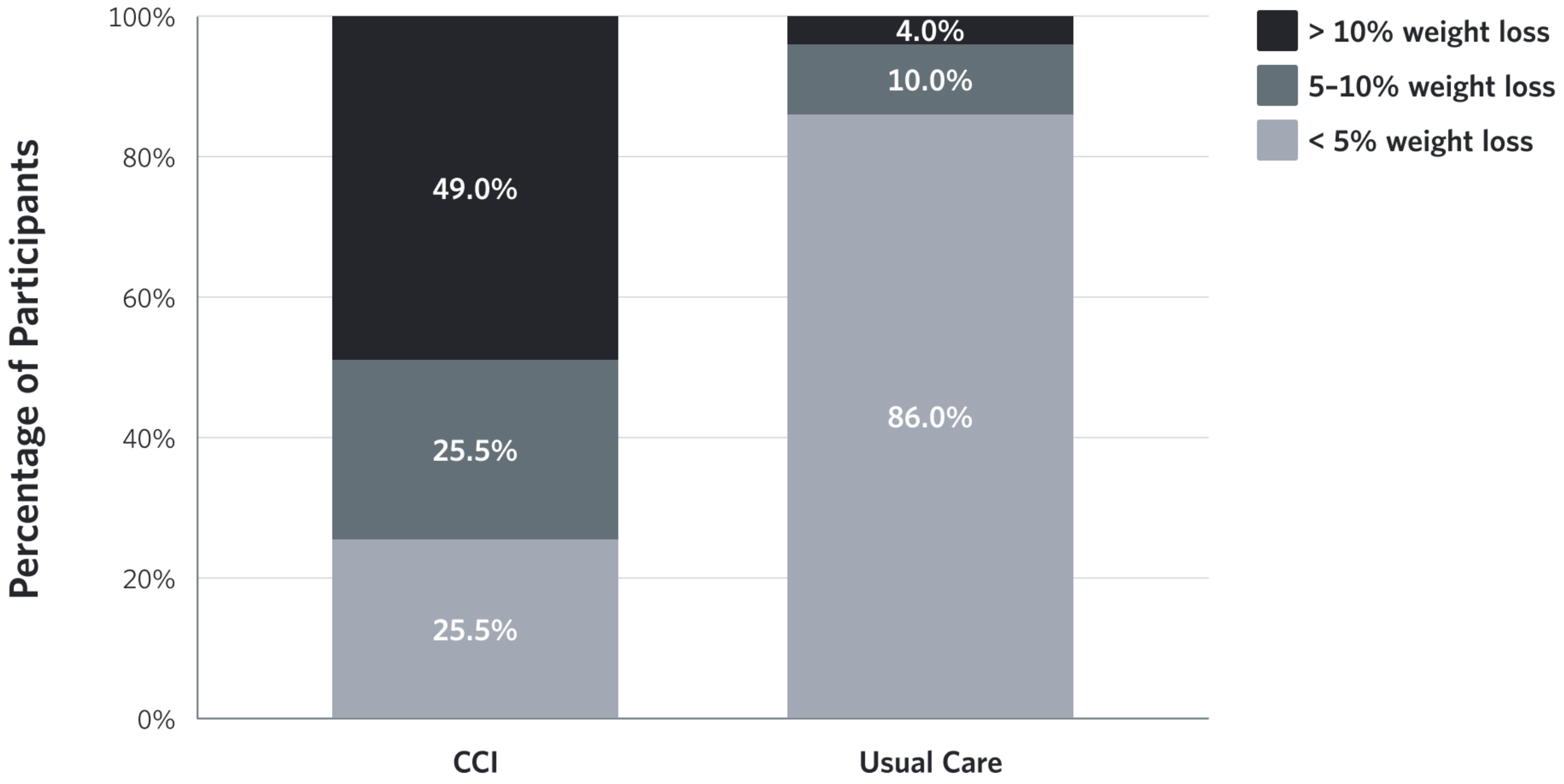

**Supplementary Figure 3**

**a**

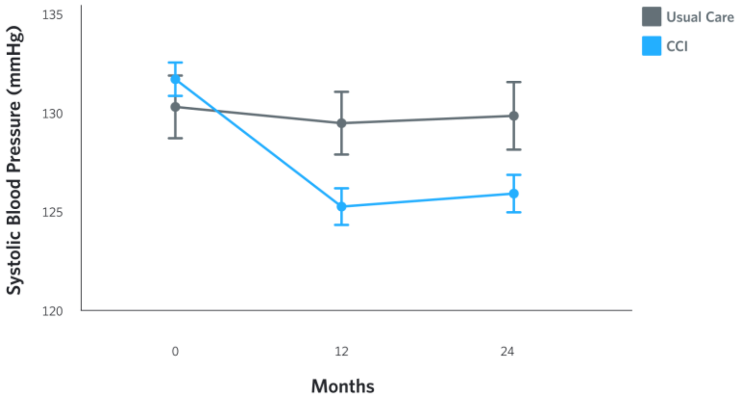


**c**

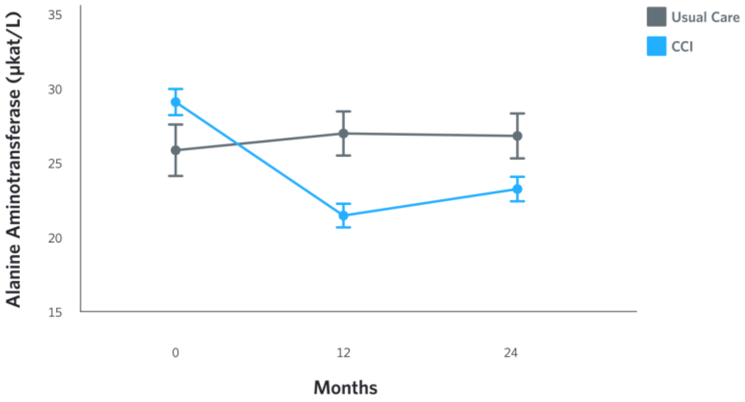


**b**

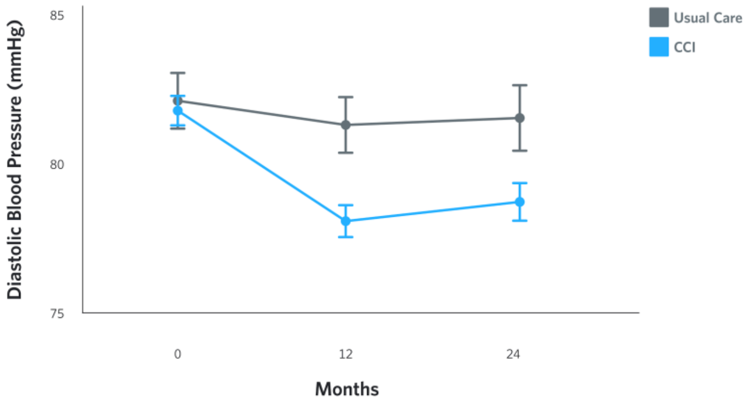


**d**

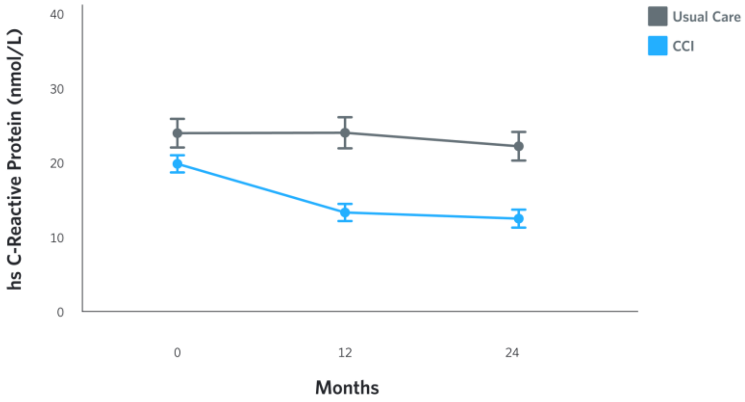

**Supplementary Figure 4**

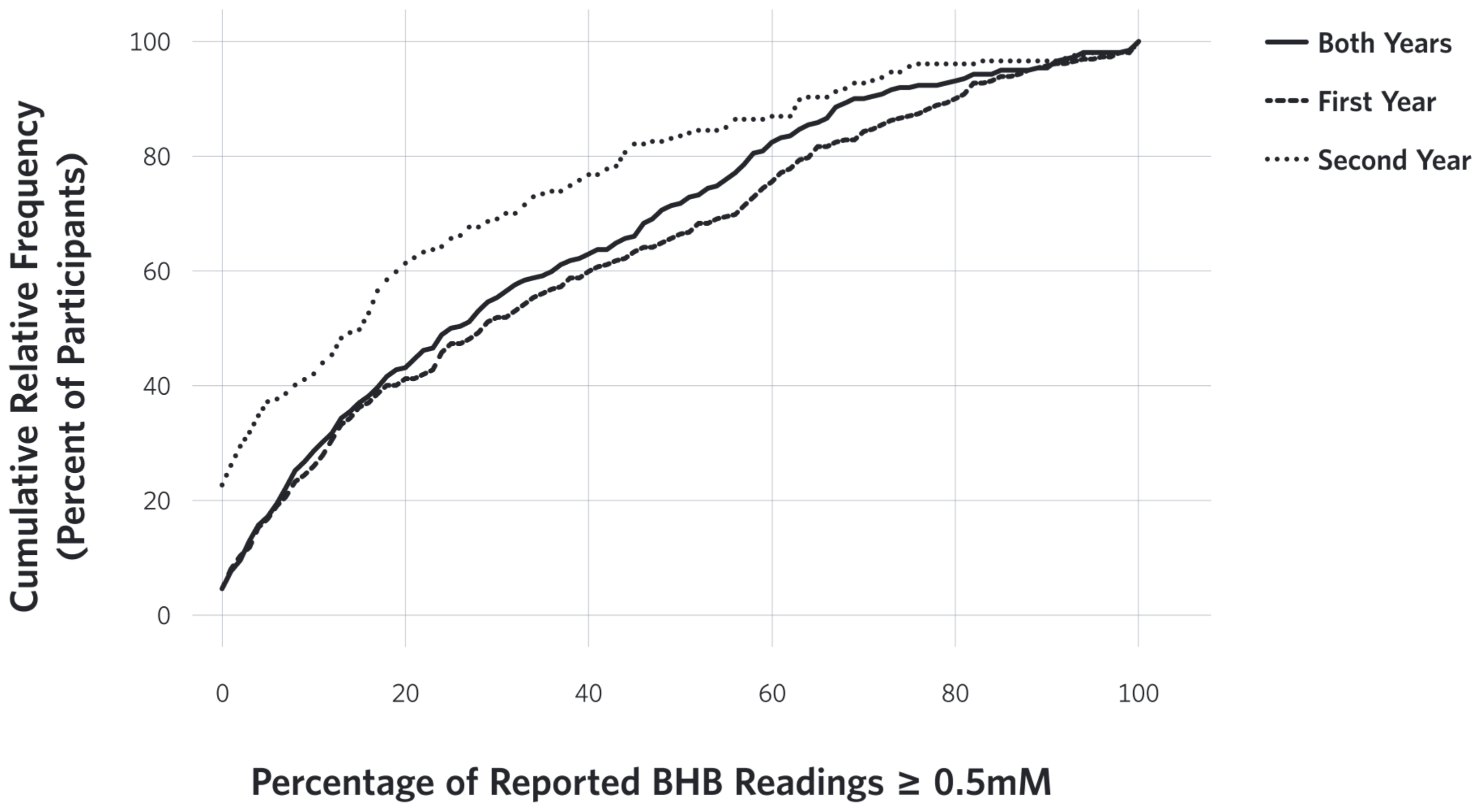

**Supplementary References (S)**


1. Buse JB, Caprio S, Cefalu WT, Ceriello A, Del Prato S, Inzucchi SE, et al. How do we define cure of diabetes? ADA Consensus Statement. Diabetes Care (2009) 32: 2133-2135.
2. International Diabetes Federation (IDF). The IDF consensus worldwide definition of the metabolic syndrome. IDF Communications (2006) 1-24.
3. Huang PL. A comprehensive definition for metabolic syndrome. Dis Model Mech (2009) 2: 231-237.
4. Kotronen A, Peltonen M, Hakkarainen A, Sevastianova K, Bergholm R, Johansson LM, et al. Prediction of non-alcoholic fatty liver disease and liver fat using metabolic and genetic factors. Gastroenterology (2009) 137: 865-872.
5. Angulo P, Hui JM, Marchesini G, Bugianesi E, George J, Farrell GC, et al. The NAFLD fibrosis score: a noninvasive system that identifies liver fibrosis in patients with NAFLD. Hepatology (2007) 45: 846-854.

TREND Statement Checklist

| Paper Section/Topic | Item No. | Descriptor | Reported? √ | Pg # |
|---|---|---|---|---|
| **TITLE and ABSTRACT** | | | | |
| Title and Abstract | 1 | • Information on how units were allocated to interventions | | 3 |
| | | • Structured abstract recommended | | 3 |
| | | • Information on target population or study sample | | 3 |
| **INTRODUCTION** | | | | |
| Background | 2 | • Scientific background and explanation of rationale | | 5-6 |
| | | • Theories used in designing behavioral interventions | | N/A |
| **METHODS** | | | | |
| Participants | 3 | • Eligibility criteria for participants, including criteria at different levels in recruitment/sampling plan (e.g., cities, clinics, subjects) | | 7,8 |
| | | • Method of recruitment (e.g., referral, self-selection), including the sampling method if a systematic sampling plan was implemented | | 7,8 |
| | | • Recruitment setting | | 7,8 |
| | | • Settings and locations where the data were collected | | 7,8 |
| Interventions | 4 | • Details of the interventions intended for each study condition and how and when they were actually administered, specifically including: | | 7,8, ref 10 |
| | | ○ Content: what was given? | | 7,8, ref 10 |
| | | ○ Delivery method: how was the content given? | | 7,8, ref 10 |
| | | ○ Unit of delivery: how were subjects grouped during delivery? | | 7,8, ref 10 |
| | | ○ Deliverer: who delivered the intervention? | | 7,8, ref 10 |
| | | ○ Setting: where was the intervention delivered? | | 7,8, ref 10 |
| | | ○ Exposure quantity and duration: how many sessions or episodes or events were intended to be delivered? How long were they intended to last? | | 7,8, ref 10 |
| | | ○ Time span: how long was it intended to take to deliver the intervention to each unit? | | 7,8, ref 10 |
| | | ○ Activities to increase compliance or adherence (e.g., incentives) | | 7,8, ref 10 |
| Objectives | 5 | • Specific objectives and hypotheses | | 6,9 |
| Outcomes | 6 | • Clearly defined primary and secondary outcome measures | | 6,9 |
| | | • Methods used to collect data and any methods used to enhance the quality of measurements | | 6,9 |

| | | | | |
|---|---|---|---|---|
| | | • Information on validated instruments such as psychometric and biometric properties | | N/A |
| Sample size | 7 | • How sample size was determined and, when applicable, explanation of any interim analyses and stopping rules | | N/A |
| Assignment method | 8 | • Unit of assignment (the unit being assigned to study condition, e.g., individual, group, community) | | 7,8, ref 10 |
| | | • Method used to assign units to study conditions, including details of any restriction (e.g., blocking, stratification, minimization) | | 7,8, ref 10 |
| | | • Inclusion of aspects employed to help minimize potential bias induced due to non-randomization (e.g., matching) | | 7,8, ref 10 |
| Blinding (masking) | 9 | • Whether or not participants, those administering the interventions, and those assessing the outcomes were blinded to study condition assignment; if so, statement regarding how the blinding was accomplished and how it was assessed | | N/A |
| Unit of Analysis | 10 | • Description of the smallest unit that is being analysed to assess intervention effects (e.g., individual, group, or community) | | 7,8, ref 10 |
| | | • If the unit of analysis differs from the unit of assignment, the analytical method used to account for this (e.g., adjusting the standard error estimates by the design effect or using multilevel analysis) | | 7,8, ref 10 |
| Statistical methods | 11 | • Statistical methods used to compare study groups for primary methods outcome(s), including complex methods for correlated data | | 11-13 |
| | | • Statistical methods used for additional analyses, such as subgroup analyses and adjusted analysis | | 11-13 |
| | | • Methods for imputing missing data, if used | | 11-13 |
| | | • Statistical software or programs used | | 11-13 |
| **RESULTS** | | | | |
| Participant flow | 12 | • Flow of participants through each stage of the study: enrollment, assignment, allocation and intervention exposure, follow-up, analysis (a diagram is strongly recommended) | | 13, Figure 1 |
| | | ○ Enrollment: the numbers of participants screened for eligibility, found to be eligible or not eligible, declined to be enrolled, and enrolled in the study | | 13, Figure 1 |
| | | ○ Assignment: the numbers of participants assigned to a study condition | | 13, Figure 1 |
| | | ○ Allocation and intervention exposure: the number of participants assigned to each study condition and the number of participants who received each intervention | | 13, Figure 1 |
| | | ○ Follow-up: the number of participants who completed the follow-up or did not complete the follow-up (i.e., lost to follow-up), by study condition | | 13, Figure 1 |
| | | ○ Analysis: the number of participants included in or excluded from the main analysis, by study condition | | 13, Figure 1 |
| | | • Description of protocol deviations from study as planned, along with reasons | | 13, Figure 1 |
| Recruitment | 13 | • Dates defining the periods of recruitment and follow-up | | 13 |
| Baseline data | 14 | • Baseline demographic and clinical characteristics of participants in each study condition | | 13, Table 1 |
| | | • Baseline characteristics for each study condition relevant to specific disease prevention research | | N/A |
| | | • Baseline comparisons of those lost to follow-up and those retained, overall and by study condition | | 13, Table 1 |

| | | | | |
|---|---|---|---|---|
| | | • Comparison between study population at baseline and target population of interest | | N/A |
| Baseline equivalence | 15 | • Data on study group equivalence at baseline and statistical methods used to control for baseline differences | | Table 1, 11-13 |
| Numbers analyzed | 16 | • Number of participants (denominator) included in each analysis for each study condition, particularly when the denominators change for different outcomes; statement of the results in absolute numbers when feasible | | Table 2, 14-18 |
| | | • Indication of whether the analysis strategy was "intention to treat" or, if not, description of how non-compliers were treated in the analyses | | Table 2, 14-18 |
| Outcomes and estimation | 17 | • For each primary and secondary outcome, a summary of results for each estimation study condition, and the estimated effect size and a confidence interval to indicate the precision | | Table 2, 14-18 |
| | | • Inclusion of null and negative findings | | Table 2, 14-18 |
| | | • Inclusion of results from testing pre-specified causal pathways through which the intervention was intended to operate, if any | | N/A |
| Ancillary analyses | 18 | • Summary of other analyses performed, including subgroup or restricted analyses, indicating which are pre-specified or exploratory | | 14-18, Suppl. Mat |
| Adverse events | 19 | • Summary of all important adverse events or unintended effects in each study condition (including summary measures, effect size estimates, and confidence intervals) | | 17,18 |
| **DISCUSSION** | | | | |
| Interpretation | 20 | • Interpretation of the results, taking into account study hypotheses, sources of potential bias, imprecision of measures, multiplicative analyses, and other limitations or weaknesses of the study | | 19-24 |
| | | • Discussion of results taking into account the mechanism by which the intervention was intended to work (causal pathways) or alternative mechanisms or explanations | | 19-24 |
| | | • Discussion of the success of and barriers to implementing the intervention, fidelity of implementation | | 19-24 |
| | | • Discussion of research, programmatic, or policy implications | | 19-24 |
| Generalizability | 21 | • Generalizability (external validity) of the trial findings, taking into account the study population, the characteristics of the intervention, length of follow-up, incentives, compliance rates, specific sites/settings involved in the study, and other contextual issues | | 19-24 |
| Overall evidence | 22 | • General interpretation of the results in the context of current evidence and current theory | | 19-24 |

*From:* Des Jarlais, D. C., Lyles, C., Crepaz, N., & the Trend Group (2004). Improving the reporting quality of nonrandomized evaluations of behavioral and public health interventions: The TREND statement. *American Journal of Public Health*, 94, 361-366. For more information, visit: http://www.cdc.gov/trendstatement/
